# Supplementary material for: Mapping the Transcriptional and Fitness Landscapes of a Pathogenic E. coli Strain: The Effects of Organic Acid Stress under Aerobic and Anaerobic Conditions
Source: Genes (Basel). 2020 Dec 31;12(1):53. doi: 10.3390/genes12010053 (PMC7824302; doi:10.3390/genes12010053)
Supplement: Supplementary file 1 [file genes-12-00053-s001.zip › genes-1011081 supplementary/genes-1011081 supplementary figures S1 - S9 HR_1.pdf]

Supplementary figures

Figure S1

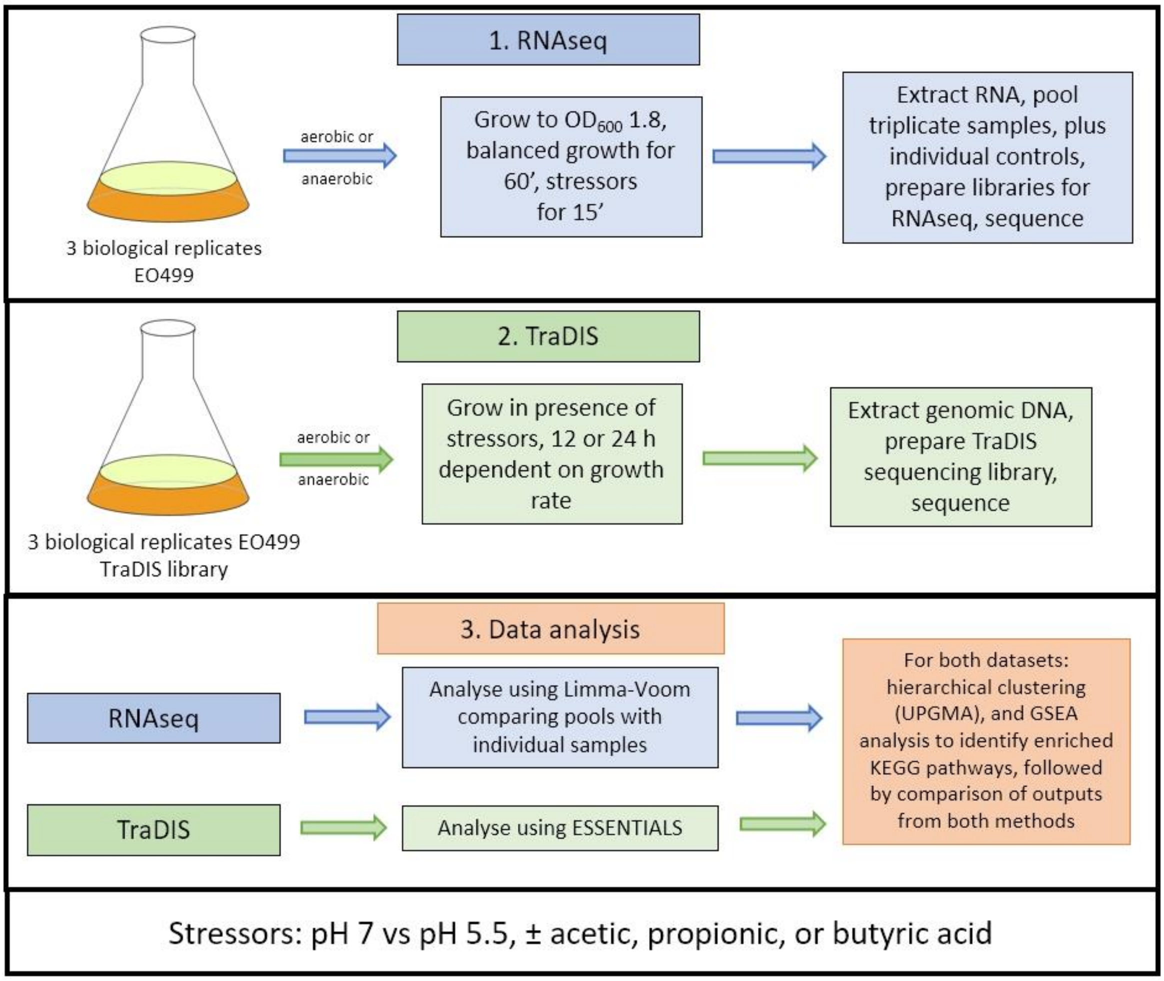

Figure S1: Summary of the experimental and data analysis steps in this study.

Figure S2

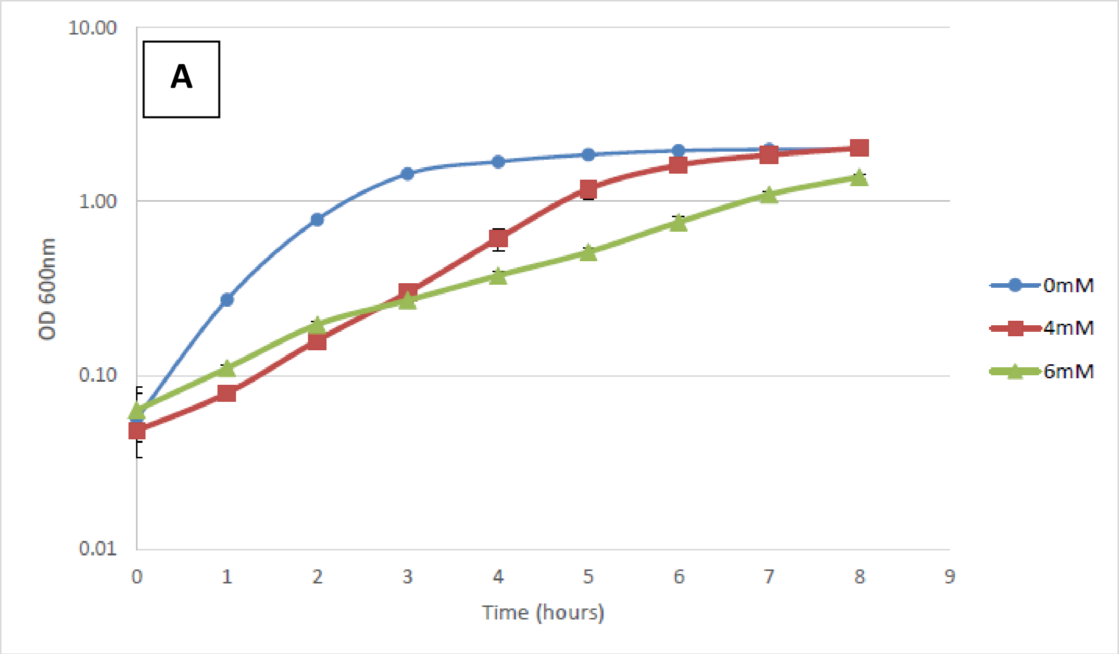

A
10.00
1.00
0.10
0.01
OD 600nm
0
1
2
3
4
5
6
7
8
9
Time (hours)
0mM
4mM
6mM

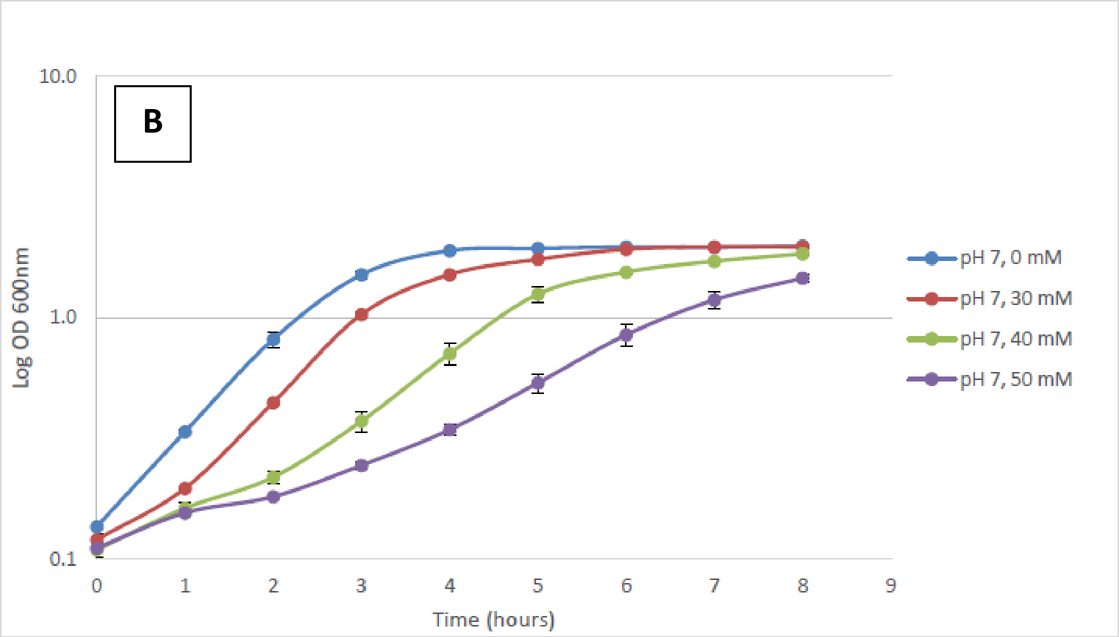

B
10.0
1.0
0.1
Log OD 600nm
0
1
2
3
4
5
6
7
8
9
Time (hours)
pH 7, 0 mM
pH 7, 30 mM
pH 7, 40 mM
pH 7, 50 mM

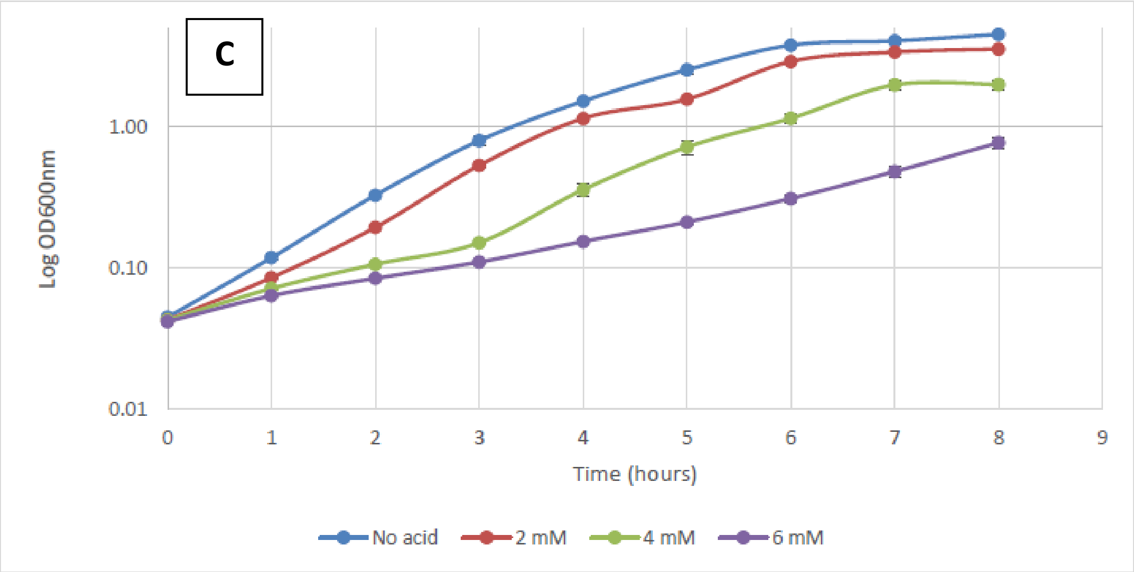
C
1.00
0.10
0.01
Log OD600nm
0
1
2
3
4
5
6
7
8
9
Time (hours)
No acid
2 mM
4 mM
6 mM

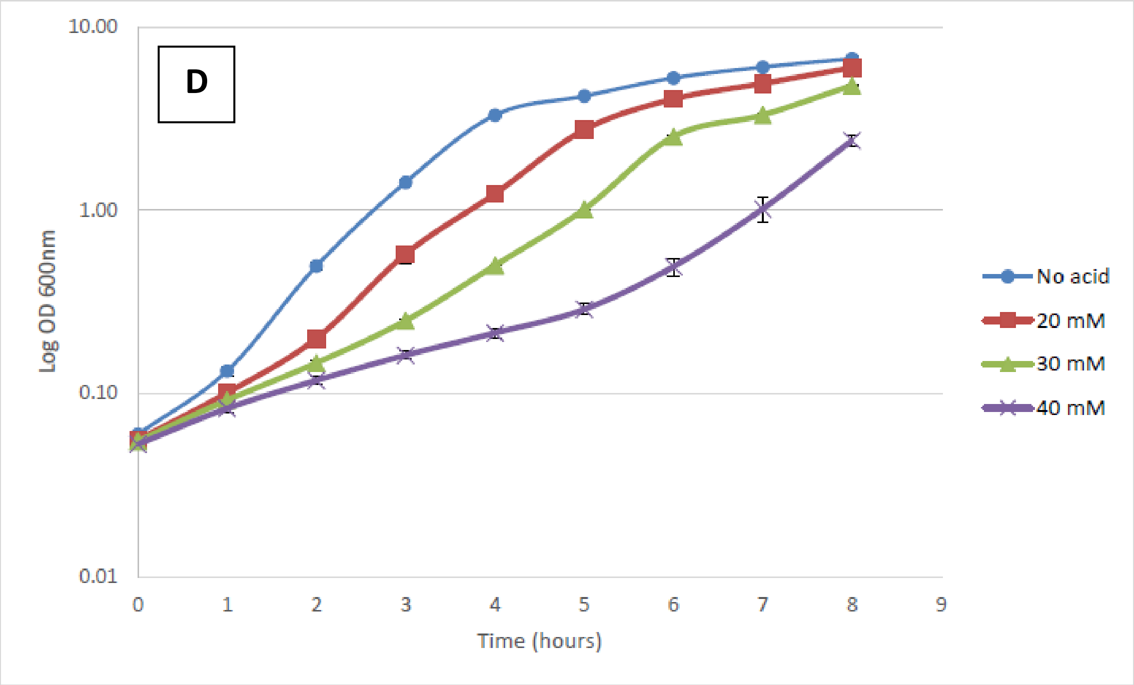
D
10.00
1.00
0.10
0.01
Log OD 600nm
0
1
2
3
4
5
6
7
8
9
Time (hours)
No acid
20 mM
30 mM
40 mM

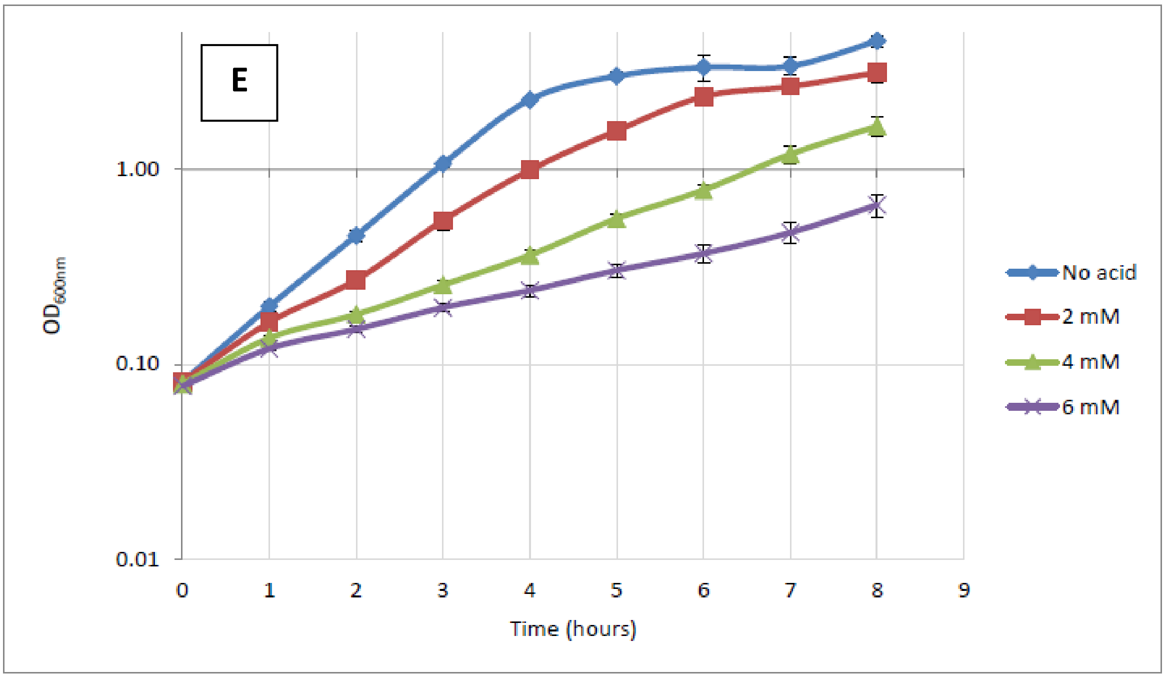

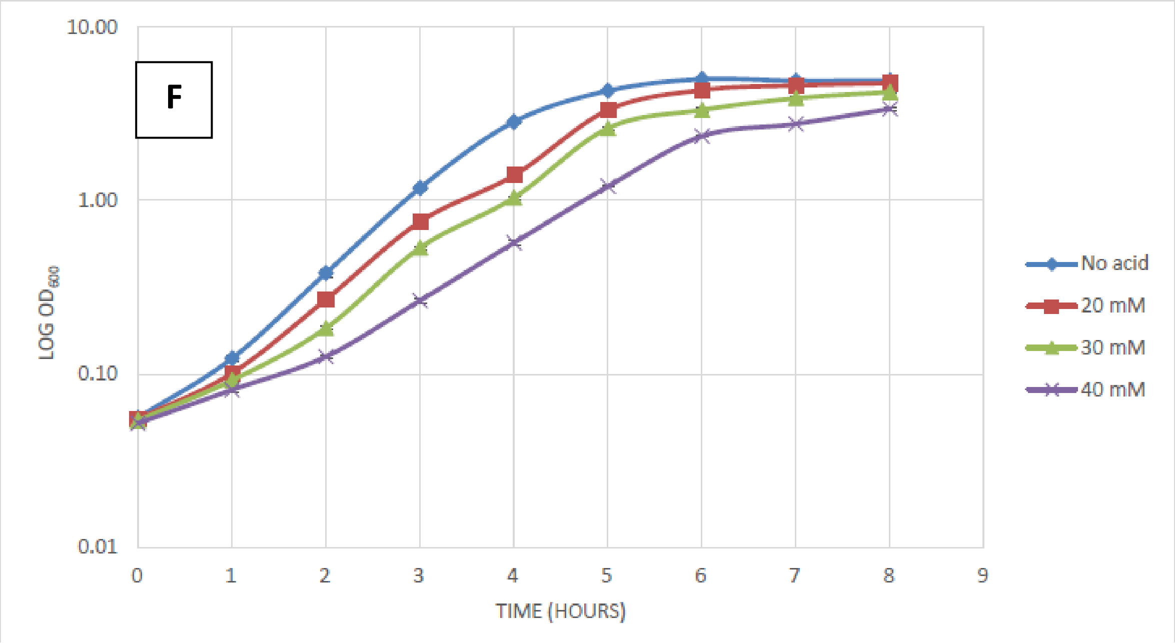

Figure S2: Affects of different SCFAs on aerobic growth rate of EO499 at pH 5.5 and pH 7. EO499 was grown in M9supp as described in Materials and Methods, in the presence of different concentrations of acetic acid (panels A, B), propionic acid (panels C, D) or butyric acid (panels E, F), at pH 5.5 (panels A, C, E) or pH 7 (panels B, D, F). Experiments were conducted in triplicate, and mean and standard deviations were plotted (error bars are not always visible as they are sometimes smaller than the symbols).

Figure S3

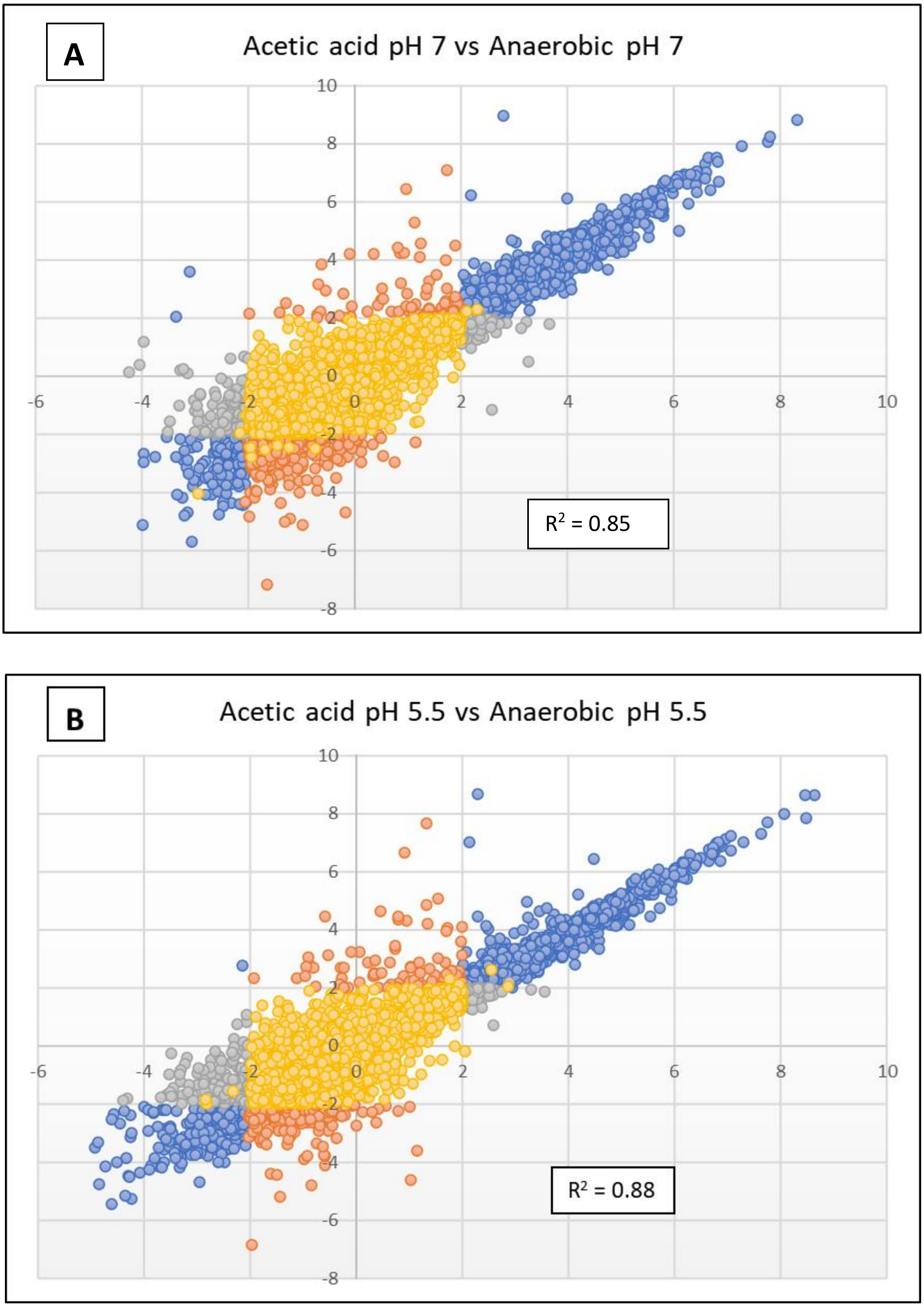

A
Acetic acid pH 7 vs Anaerobic pH 7
10
8
6
4
2
0
-2
-4
-6
-8
-6
-4
-2
0
2
4
6
8
10
R^2 = 0.85
B
Acetic acid pH 5.5 vs Anaerobic pH 5.5
10
8
6
4
2
0
-2
-4
-6
-8
-6
-4
-2
0
2
4
6
8
10
R^2 = 0.88

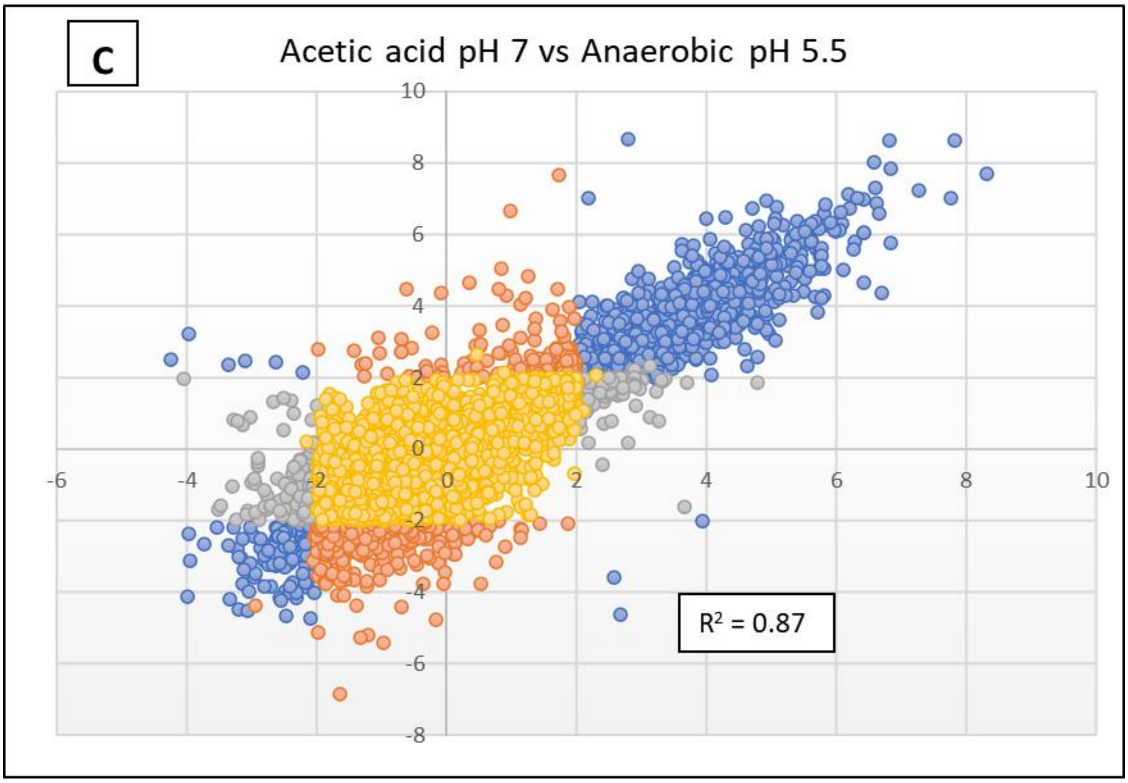

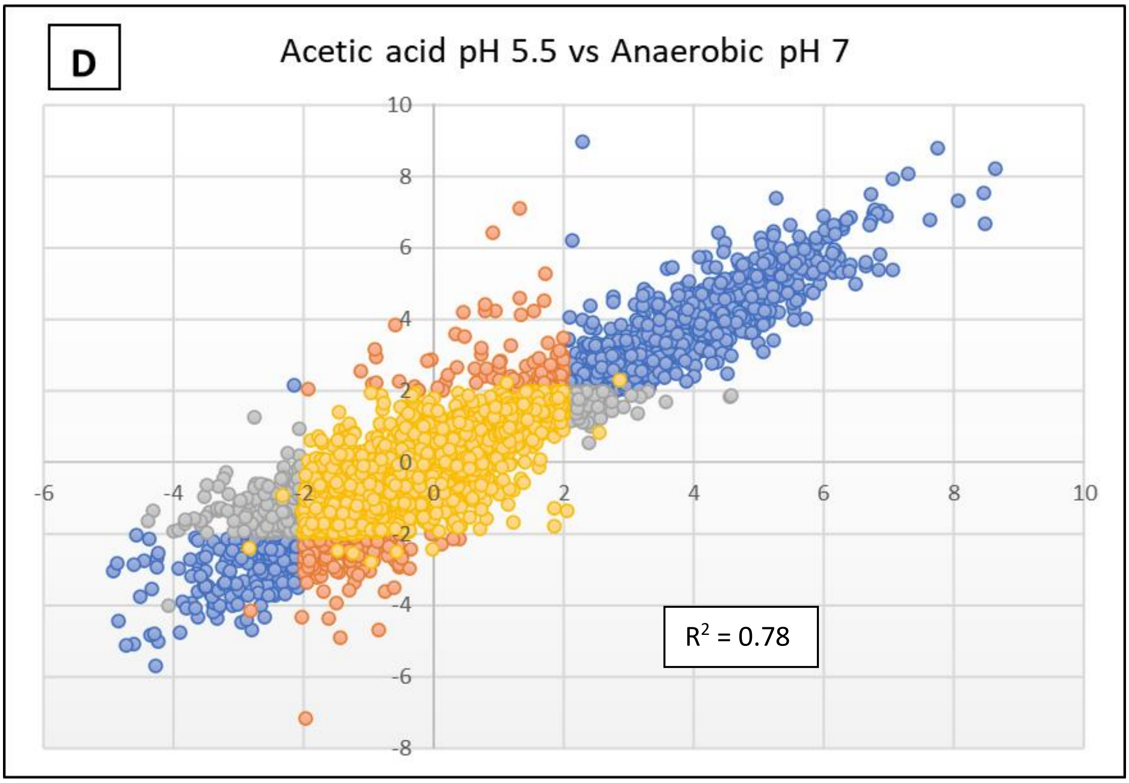

Figure S3: Correlations of gene expression levels between aerobic cells exposed to acetic acid, and cells grown anaerobically. Horizontal axis: log2-fold change in expression on addition of acetic acid at stated pH; vertical axis: log2-fold change in expression anaerobic/aerobic at stated pH. Blue symbols: change significant for both conditions; orange symbols: change significant for acetic acid treated cells only; grey symbols: change

significant for anaerobic cells only; yellow symbols: change not significant. Significant cut-off taken at log2-fold change is taken as >2 or <-2, adjusted p value <0.05.

Figure S4

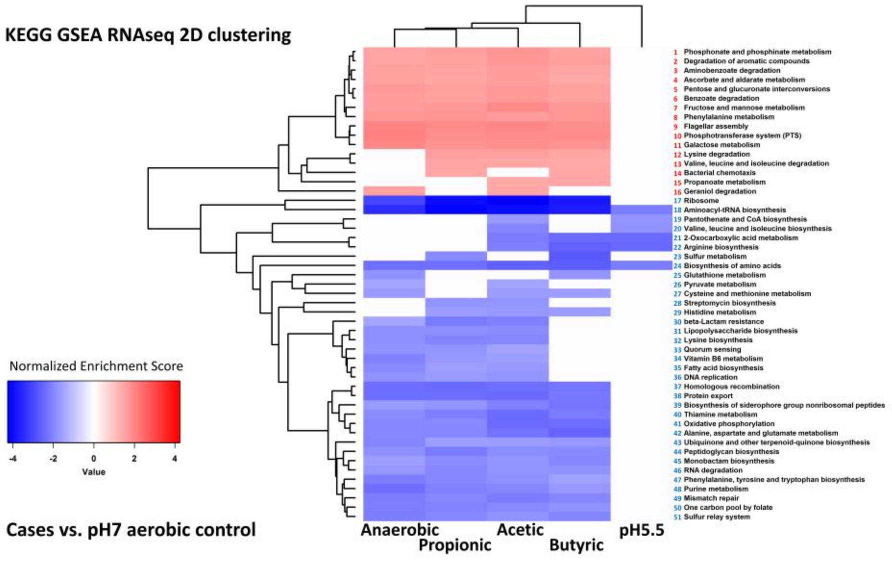

Figure S4A

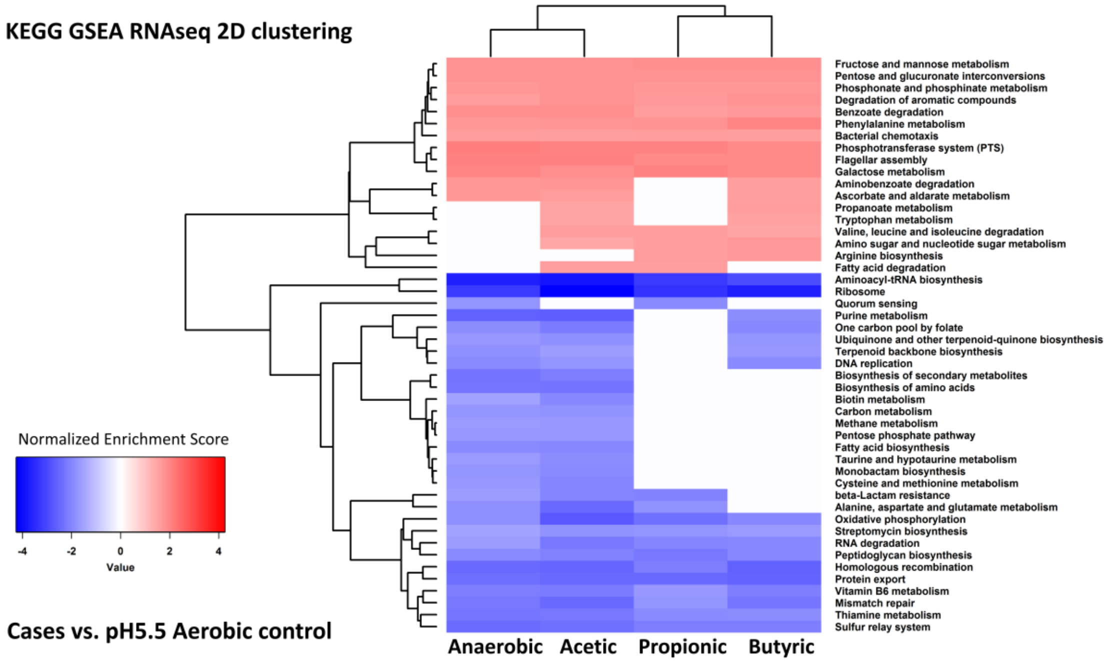

Figure S4B

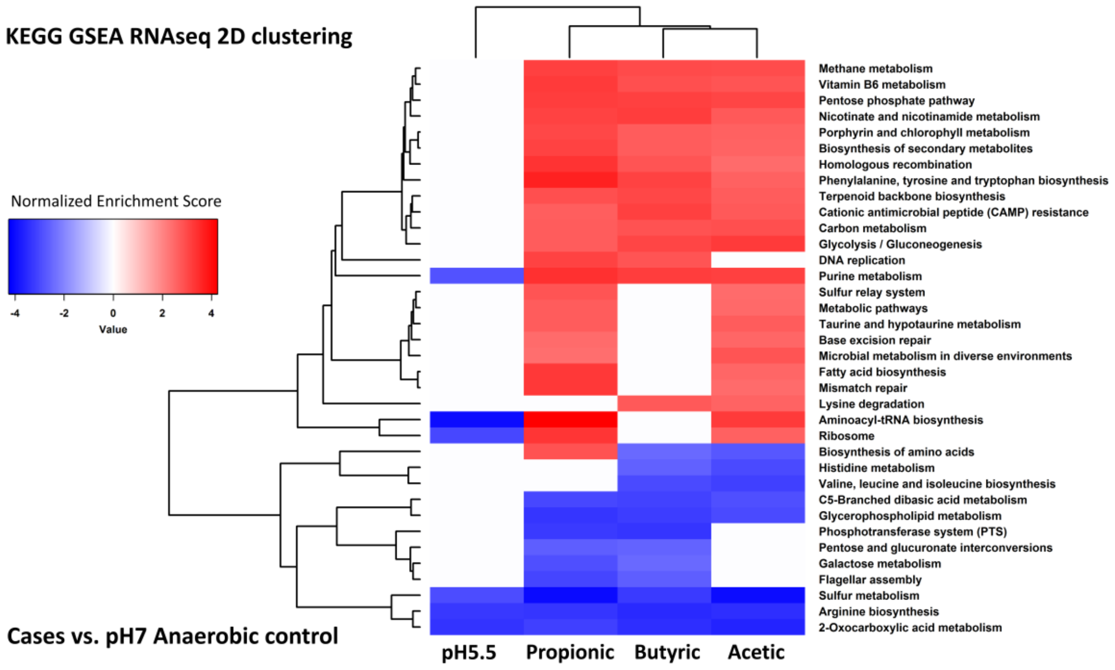

Figure S4C

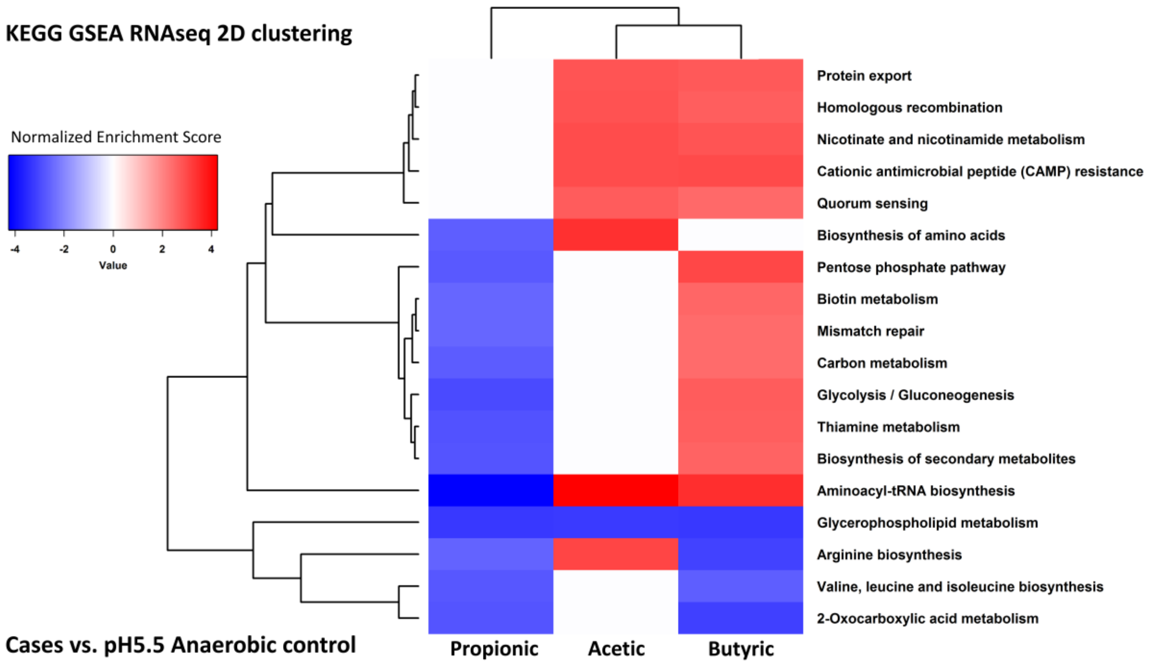

Figure S4D

Figure S4: Clustering of KEGG pathways enriched in GSEA analysis of RNAseq data.

Figure S5

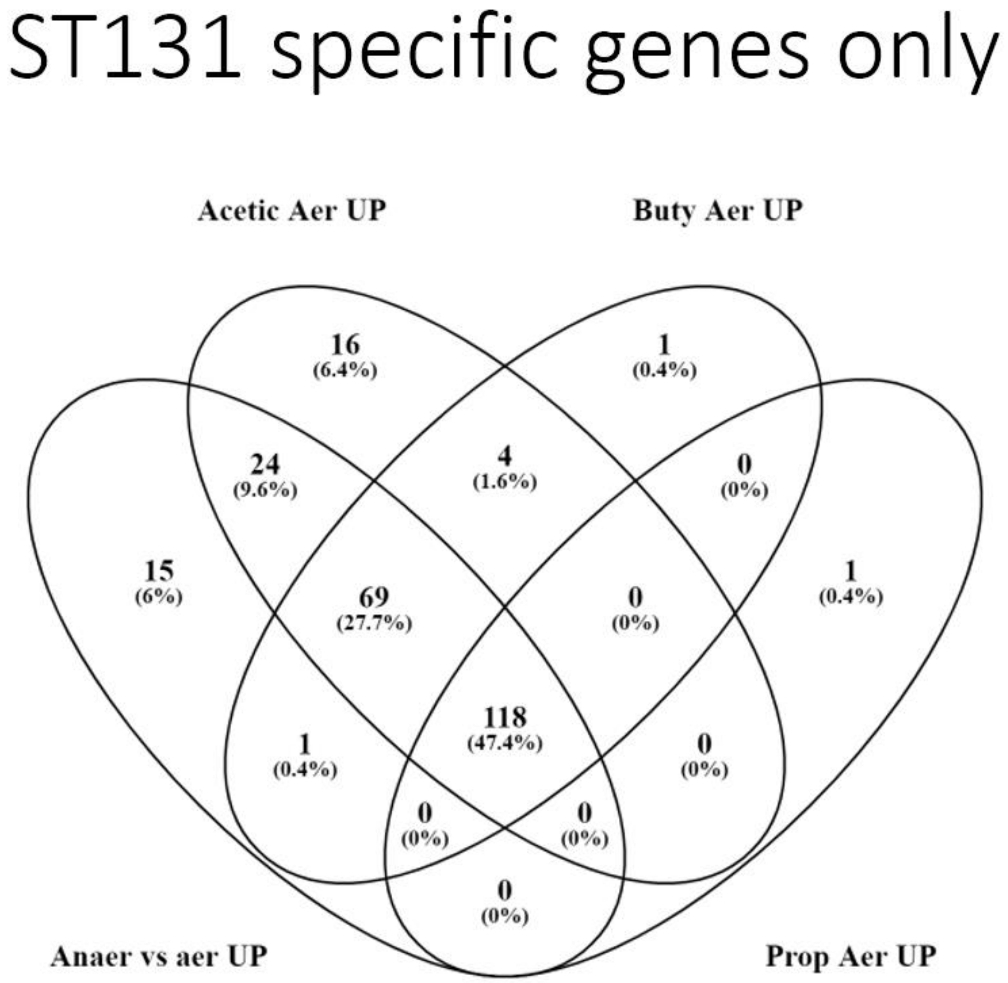

Figure S5A

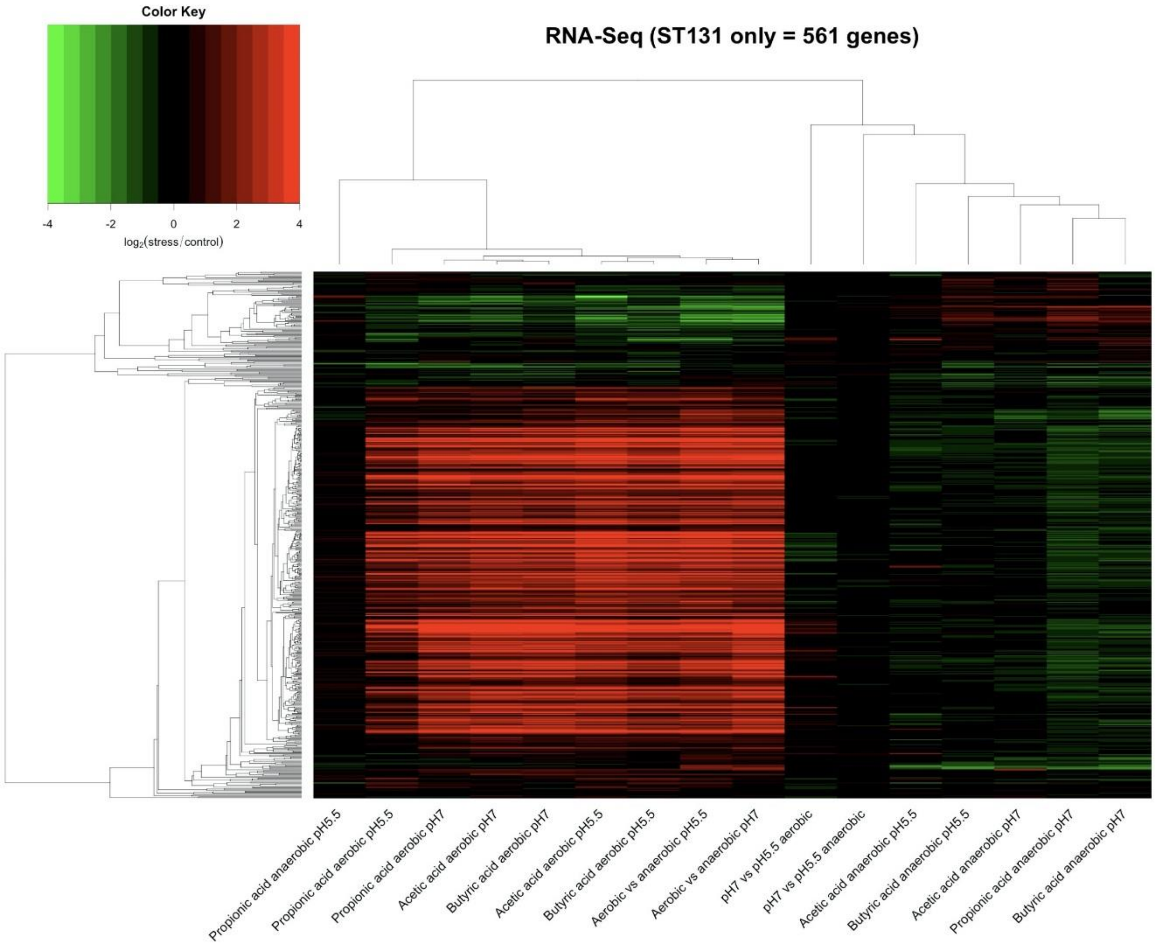

Figure S5B

Figure S5: Many genes regulated by SCFAs and anaerobic growth are not part of the *E. coli* core genome. The Venn diagram (S4A) shows genes annotated as ST131-specific (and are therefore "non-core") that were significantly up-regulated (log_2fold change >2, adjusted P value < 0.05) under the conditions shown irrespective of pH. The relative expression levels of all non-core genes are shown in the cluster analysis (S4B), with conditions labelled along the bottom; all comparisons are with the control lacking organic acid, or as labelled.

Figure S6

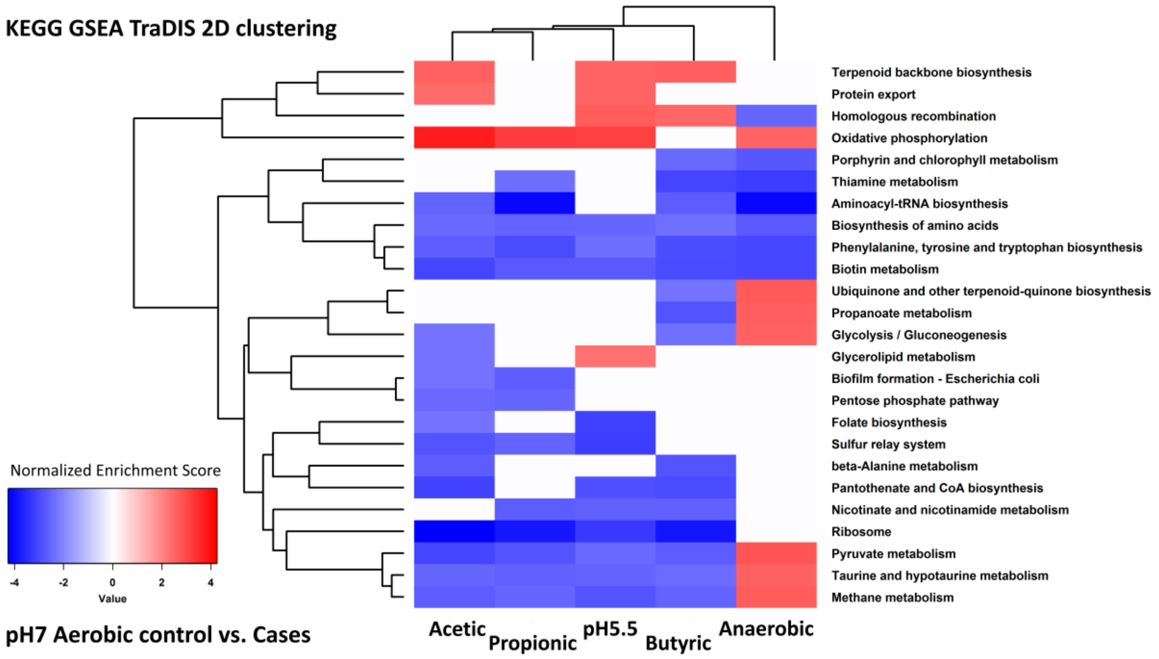

Figure S6A

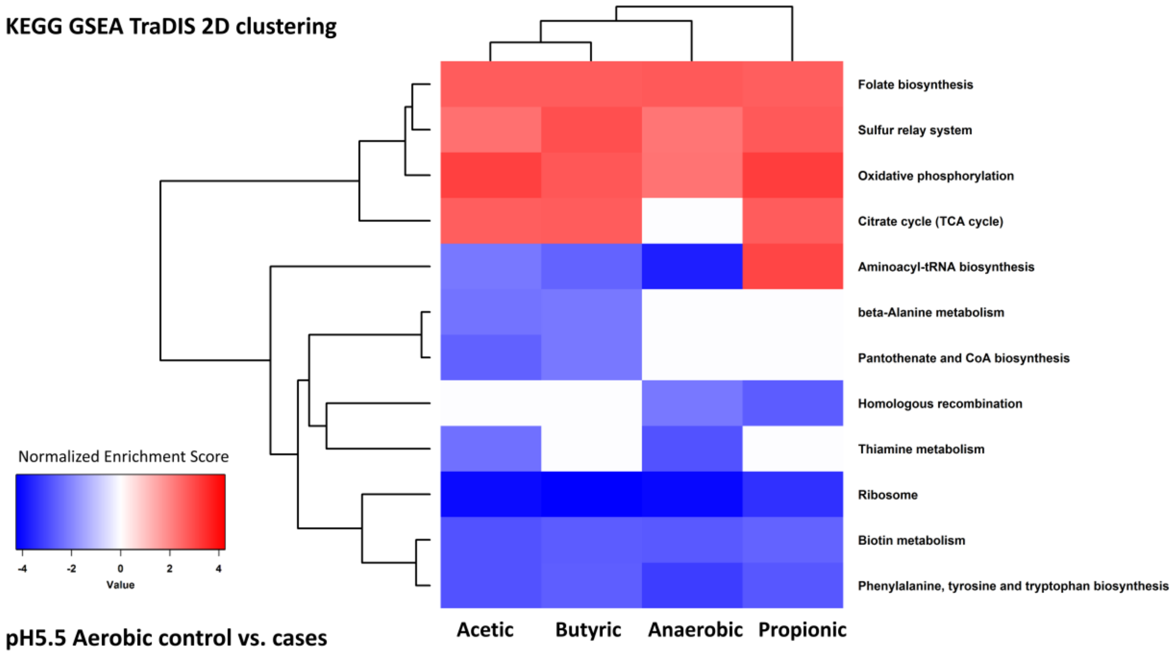

Figure S6B

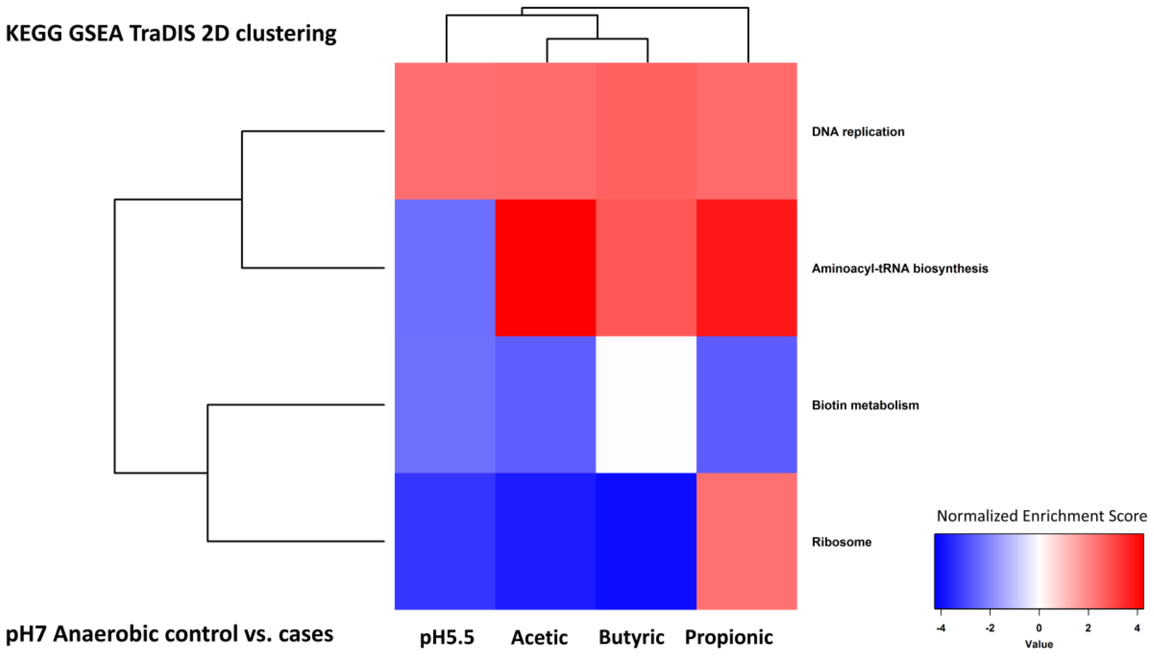

Figure S6C

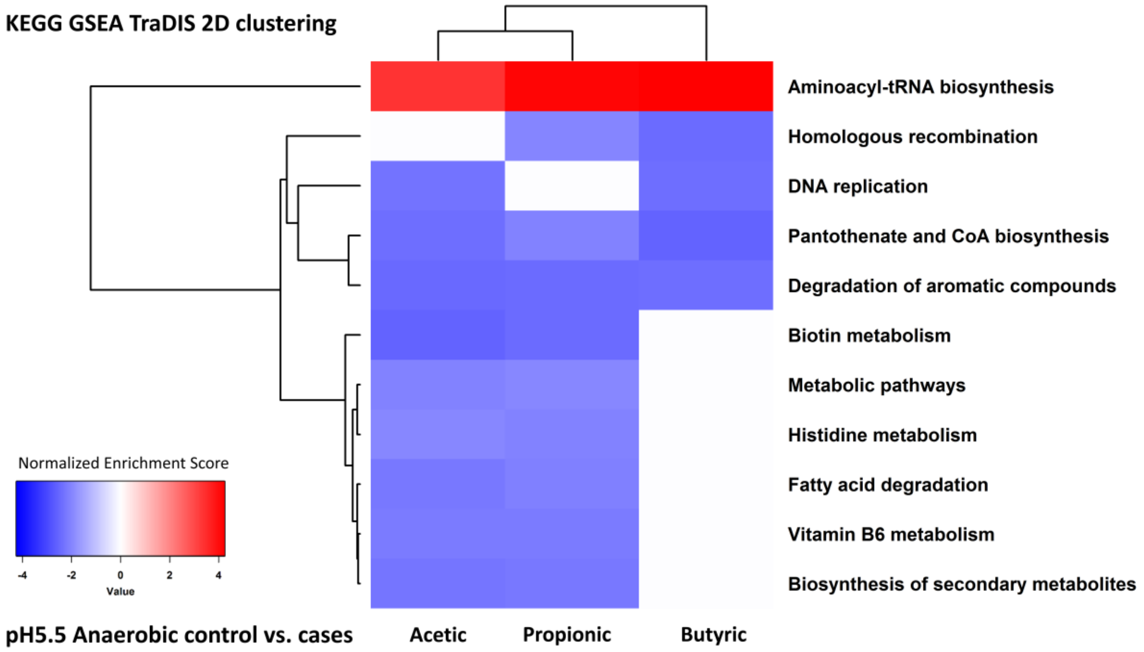

Figure S6D

Figure S6. Clustering of KEGG pathways identified from TraDIS data by Gene Set Enrichment Analysis. TraDIS data were analysed and clustered as described in Materials and Methods. In each

of the figures, comparisons are with the relevant control without SCFA, as follows: pH 7 aerobic (A), pH 5.5 aerobic (B), pH 7 anaerobic (C), pH 5.5 anaerobic (D). Only pathways which showed significant changes in at least two conditions are shown. Clusters in red are those where mutations generally cause reduced fitness in the experimental condition compared to the control condition; clusters in blue are those where mutations generally cause reduced fitness in the control condition compared to the experimental condition.

Figure S7

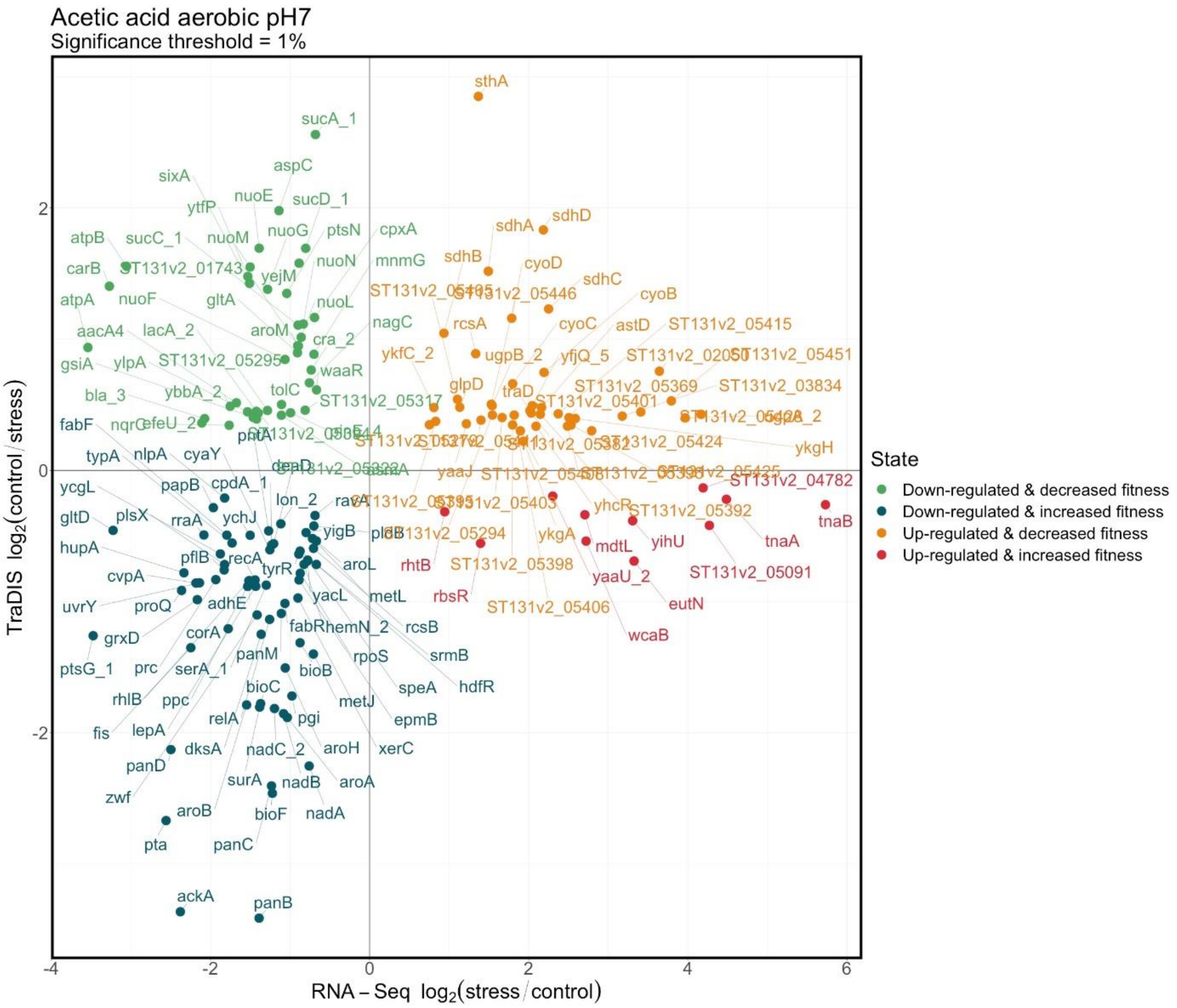

Figure S7A

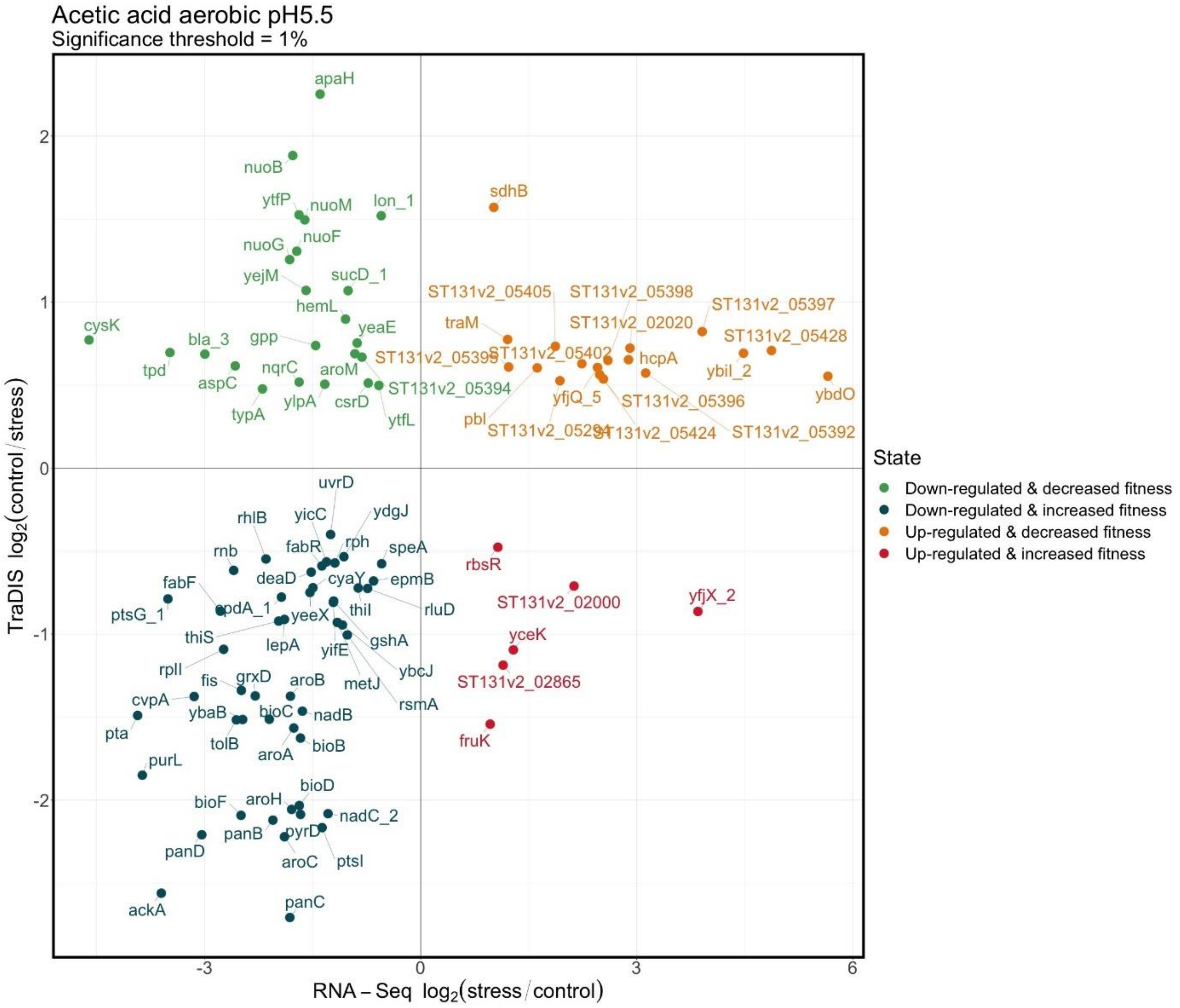

Acetic acid aerobic pH5.5
Significance threshold = 1%
TraDIS log2(control/stress)
RNA – Seq log2(stress/control)
2
1
0
-1
-2
-3
0
3
6
apaH
nuoB
ytfP
nuoM
lon_1
nuoG
nuoF
yejM
sucD_1
hemL
cysK
bla_3
gpp
yeaE
tpd
aspC
nqrC
aroM
typA
ylpA
csrD
ytfL
ST131v2_05394
sdhB
ST131v2_05405
ST131v2_05398
ST131v2_05397
traM
ST131v2_02020
ST131v2_05428
ST131v2_05399
ST131v2_05402
hcpA
ybiI_2
yfjQ_5
ybdO
pbl
ST131v2_05396
ST131v2_05294
ST131v2_05424
ST131v2_05392
State
Down-regulated & decreased fitness
Down-regulated & increased fitness
Up-regulated & decreased fitness
Up-regulated & increased fitness
uvrD
rhlB
yicC
ydgJ
rnb
fabR
rph
speA
deaD
cyaY
epmB
fabF
rbsR
ptsG_1
cpdA_1
yeeX
thiI
rluD
ST131v2_02000
yfjX_2
thiS
lepA
yifE
gshA
yceK
rplI
ybcJ
fis
grxD
aroB
metJ
ST131v2_02865
cvpA
bioC
nadB
rsmA
pta
ybaB
tolB
aroA
bioB
fruK
purL
bioD
bioF
aroH
nadC_2
panB
pyrD
panD
aroC
ptsI
ackA
panC

Figure S7B

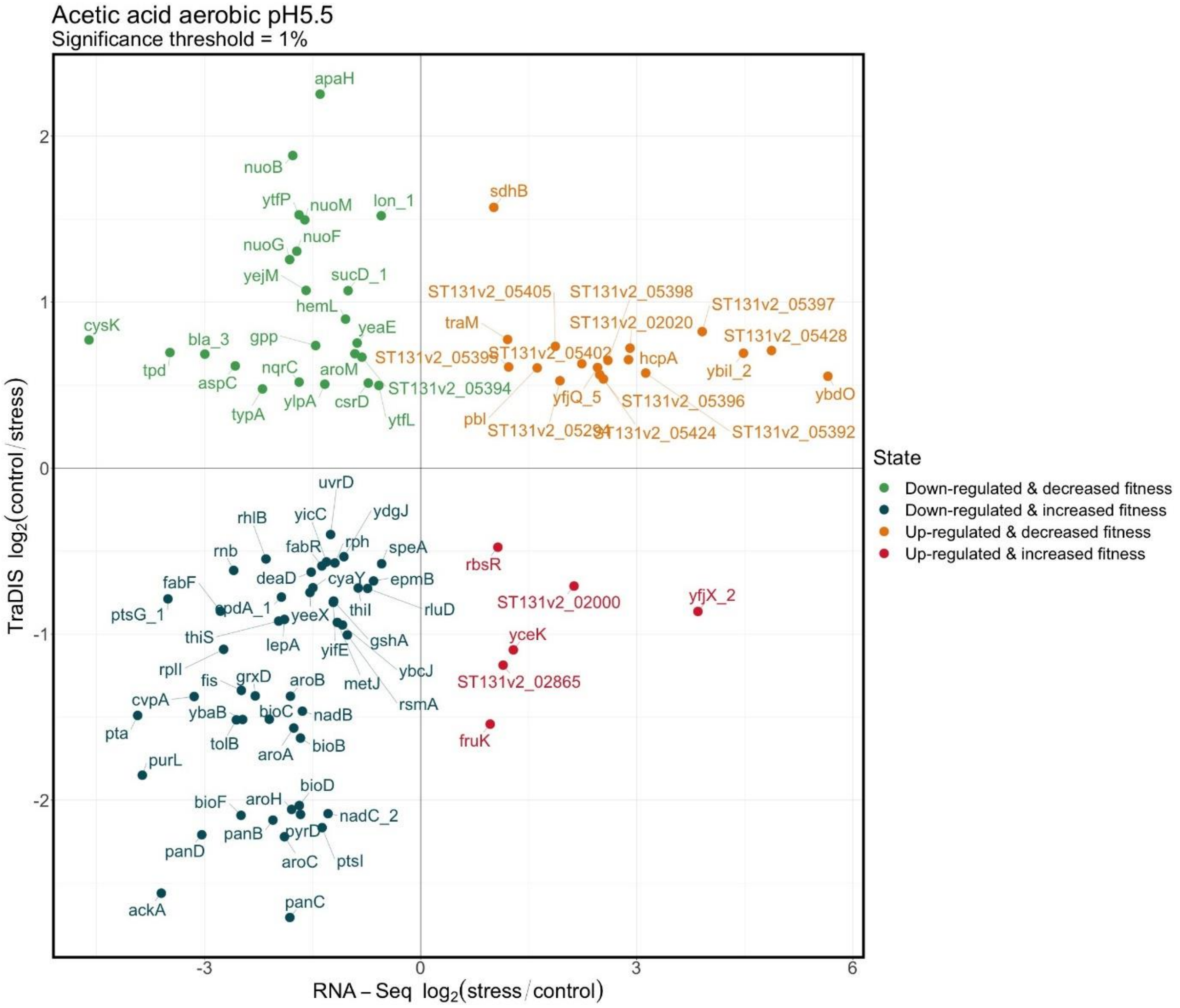
Acetic acid aerobic pH5.5
Significance threshold = 1%
TraDIS log2(control / stress)
RNA – Seq log2(stress / control)
State
Down-regulated & decreased fitness
Down-regulated & increased fitness
Up-regulated & decreased fitness
Up-regulated & increased fitness
apaH
nuoB
ytfP
nuoM
lon_1
nuoG
nuoF
yejM
sucD_1
hemL
cysK
bla_3
gpp
yeaE
tpd
aspC
nqrC
aroM
typA
ylpA
csrD
ytfL
ST131v2_05394
sdhB
ST131v2_05405
ST131v2_05398
ST131v2_05397
traM
ST131v2_02020
ST131v2_05428
hcpA
ybil_2
ybdO
yfjQ_5
ST131v2_05396
pbl
ST131v2_05424
ST131v2_05392
uvrD
rhlB
yicC
ydgJ
rnb
fabR
rph
speA
deaD
cyaY
epmB
fabF
thiI
rluD
ptsG_1
yeeX
gshA
thiS
lepA
yifE
ybcJ
rplI
fis
grxD
aroB
metJ
rsmA
cvpA
ybaB
bioC
nadB
pta
tolB
aroA
bioB
purL
bioF
aroH
bioD
nadC_2
panB
pyrD
panD
aroC
ptsI
ackA
panC
rbsR
ST131v2_02000
yfjX_2
yceK
ST131v2_02865
fruK

Figure S7C

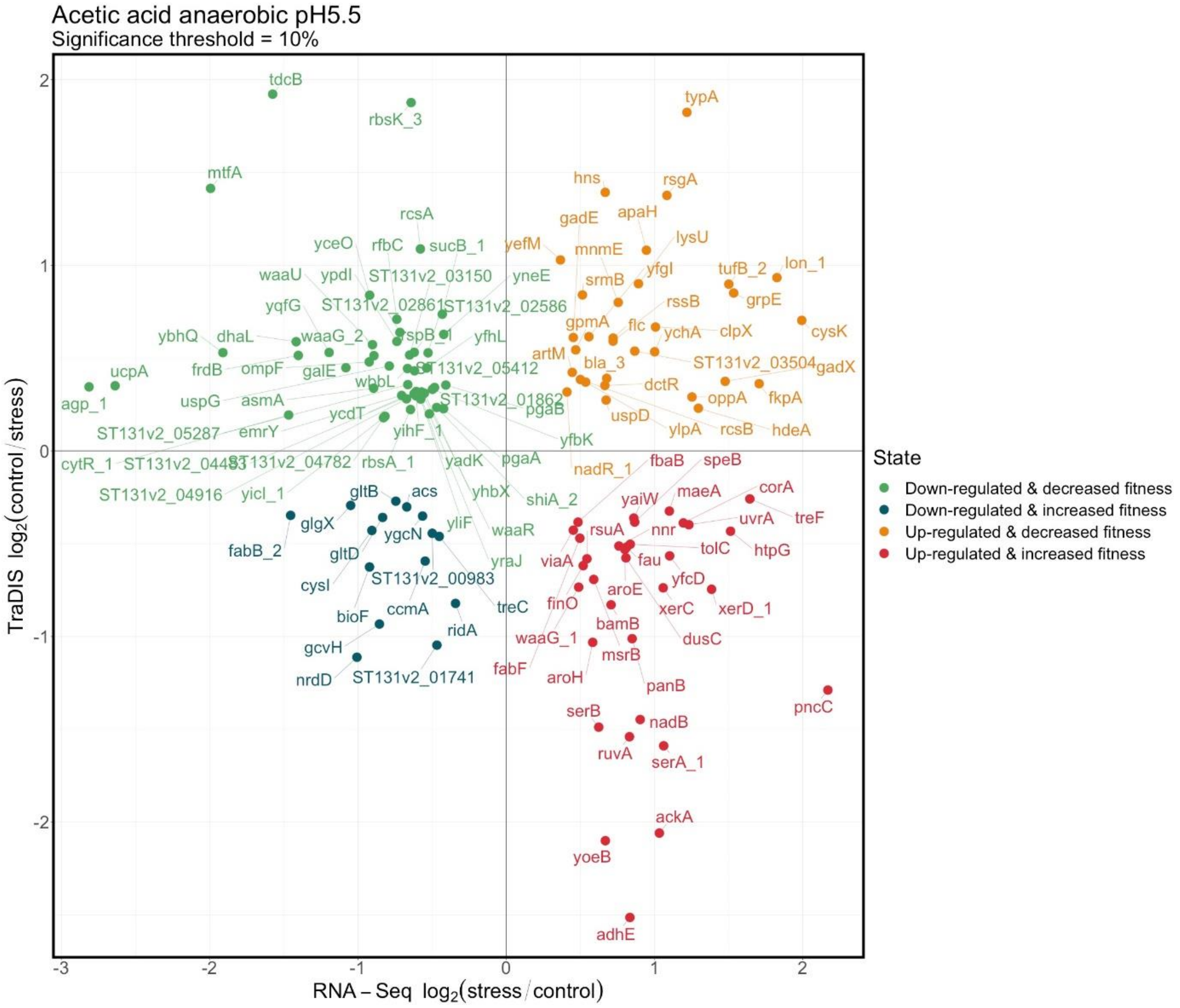

Acetic acid anaerobic pH5.5
Significance threshold = 10%
TraDIS log2(control/stress)
RNA – Seq log2(stress/control)
State
Down-regulated & decreased fitness
Down-regulated & increased fitness
Up-regulated & decreased fitness
Up-regulated & increased fitness

Figure S7D

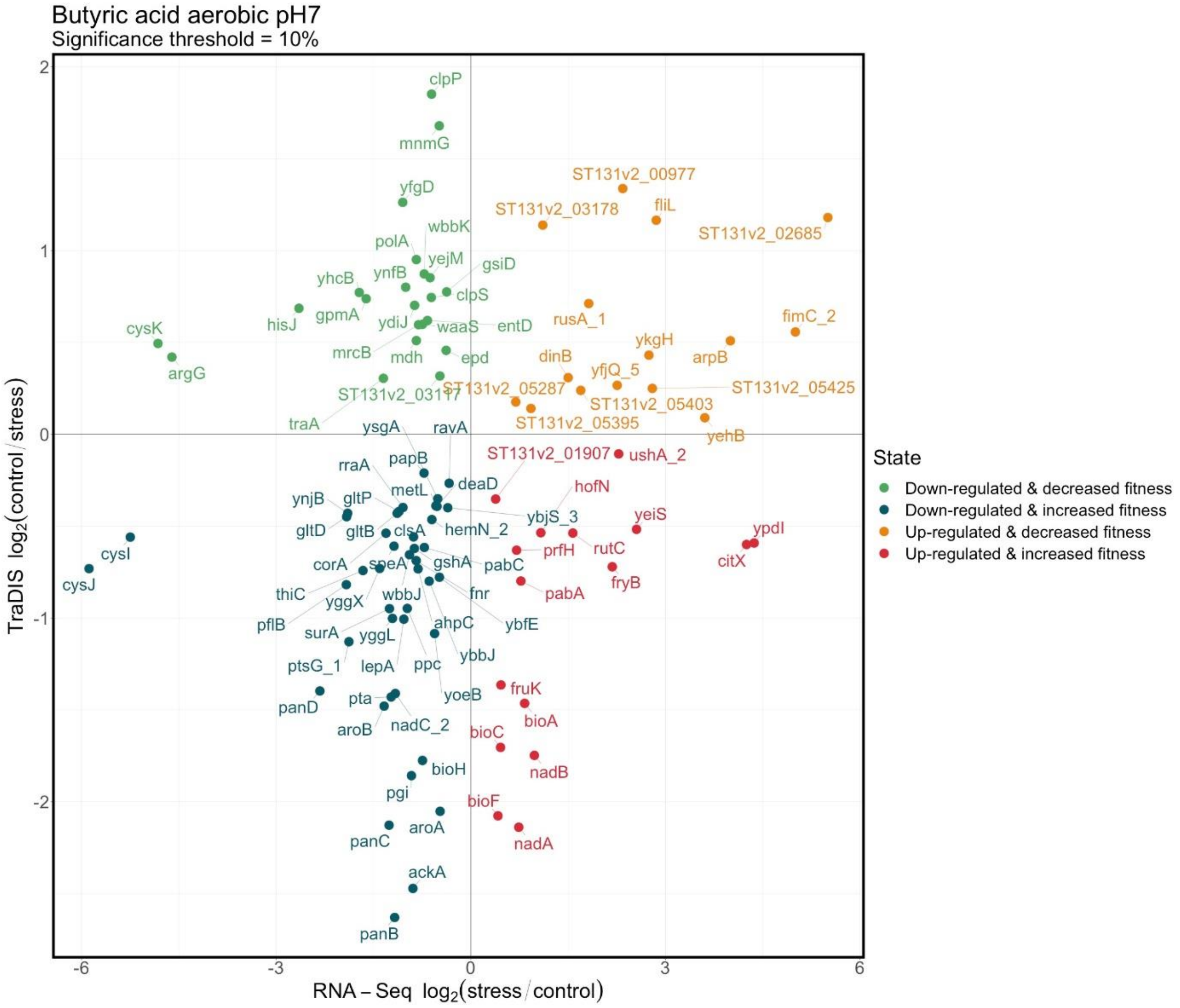
Butyric acid aerobic pH7
Significance threshold = 10%
TraDIS log2(control/stress)
RNA – Seq log2(stress/control)
State
Down-regulated & decreased fitness
Down-regulated & increased fitness
Up-regulated & decreased fitness
Up-regulated & increased fitness

Figure S7E

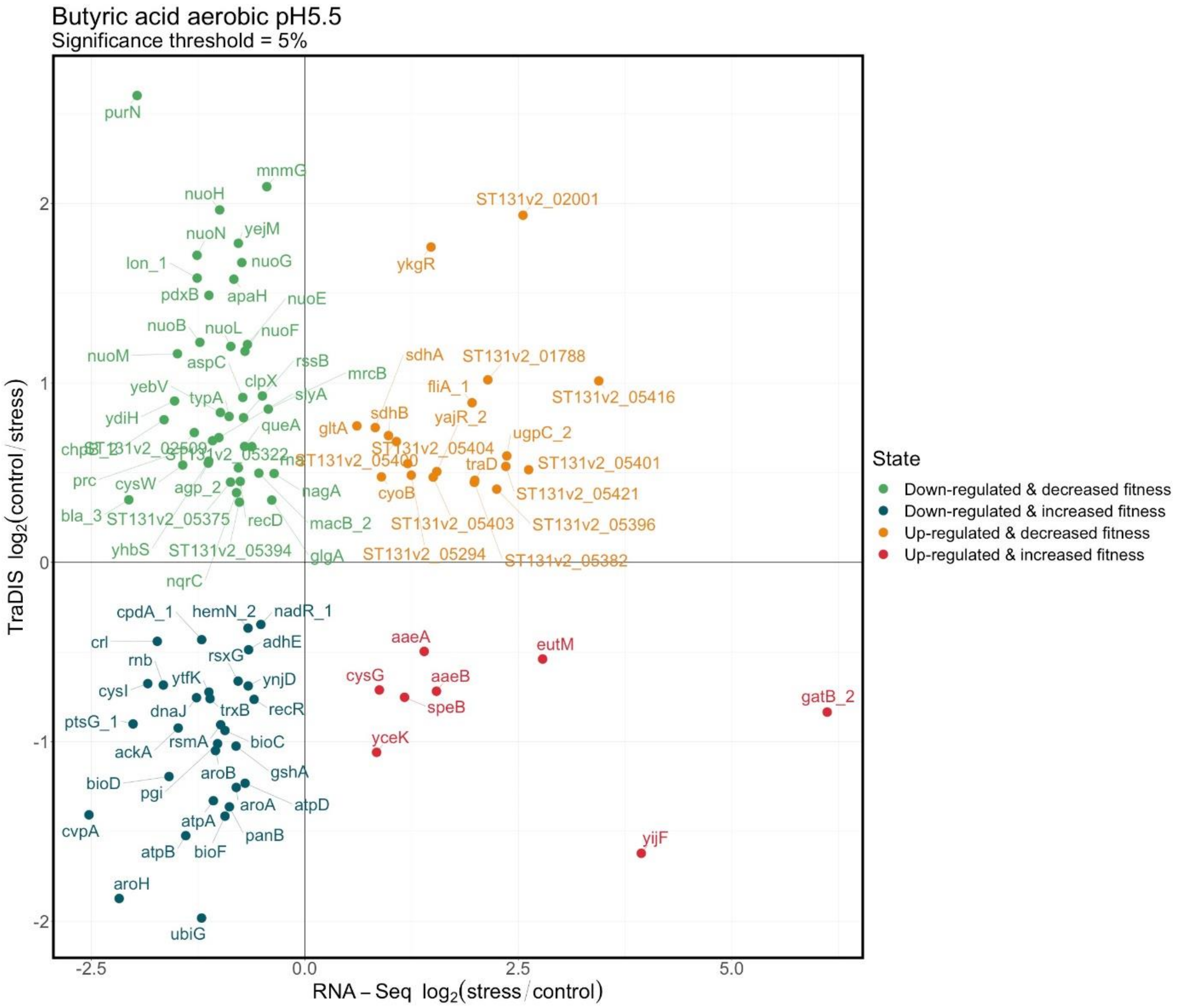

Butyric acid aerobic pH5.5
Significance threshold = 5%
TraDIS log2(control/stress)
RNA – Seq log2(stress/control)
State
Down-regulated & decreased fitness
Down-regulated & increased fitness
Up-regulated & decreased fitness
Up-regulated & increased fitness
purN
mnmG
nuoH
yejM
nuoN
nuoG
lon_1
pdxB
apaH
nuoE
nuoB
nuoL
nuoF
nuoM
aspC
rssB
mrcB
yebV
clpX
typA
slyA
ydiH
queA
prc
cysW
agp_2
nagA
bla_3
ST131v2_05375
recD
macB_2
yhbS
ST131v2_05394
glgA
nqrC
ST131v2_02001
ykgR
sdhA
ST131v2_01788
fliA_1
ST131v2_05416
sdhB
yajR_2
gltA
ugpC_2
ST131v2_05404
traD
ST131v2_05401
cyoB
ST131v2_05421
ST131v2_05403
ST131v2_05396
ST131v2_05294
ST131v2_05382
cpdA_1
hemN_2
nadR_1
crl
adhE
rnb
rsxG
ytfK
ynjD
cysI
dnaJ
trxB
recR
ptsG_1
ackA
rsmA
bioC
gshA
bioD
aroB
pgi
aroA
atpD
cvpA
atpA
panB
atpB
bioF
aroH
ubiG
aaeA
eutM
cysG
aaeB
speB
gatB_2
yceK
yijF

Figure S7F

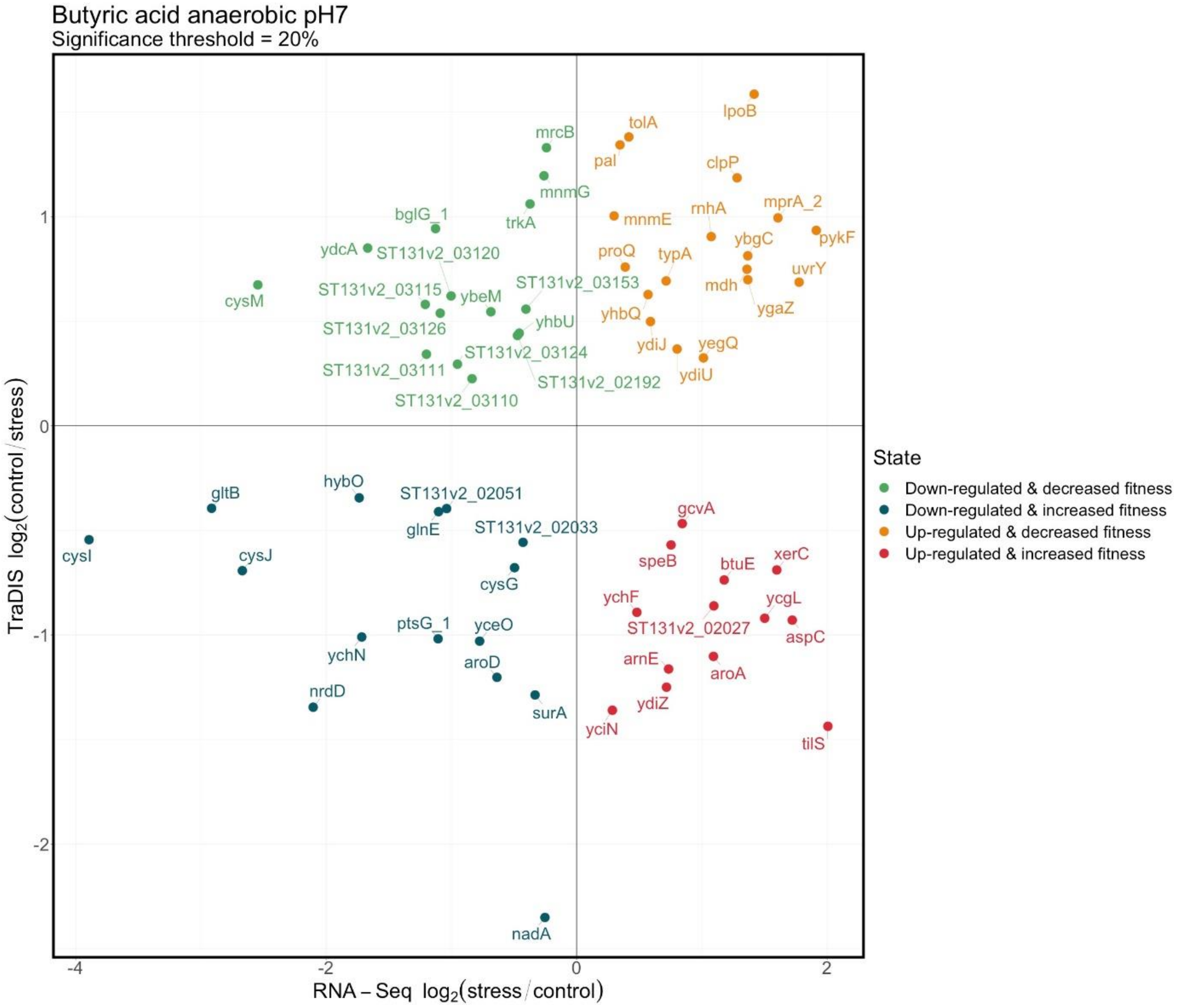

Butyric acid anaerobic pH7
Significance threshold = 20%
TraDIS log2(control/stress)
RNA – Seq log2(stress/control)
State
Down-regulated & decreased fitness
Down-regulated & increased fitness
Up-regulated & decreased fitness
Up-regulated & increased fitness
mrcB
mnmG
trkA
bglG_1
ydcA
ST131v2_03120
cysM
ST131v2_03115
ybeM
ST131v2_03153
ST131v2_03126
yhbU
ST131v2_03124
ST131v2_03111
ST131v2_02192
ST131v2_03110
tolA
pal
lpoB
clpP
rnhA
mprA_2
mnmE
ybgC
pykF
proQ
typA
uvrY
mdh
ygaZ
yhbQ
ydiJ
yegQ
ydiU
gltB
hybO
ST131v2_02051
glnE
ST131v2_02033
cysI
cysJ
cysG
ptsG_1
yceO
ychN
aroD
nrdD
surA
nadA
gcvA
speB
btuE
xerC
ychF
ycgL
ST131v2_02027
aspC
arnE
aroA
ydiZ
yciN
tilS

Figure S7G

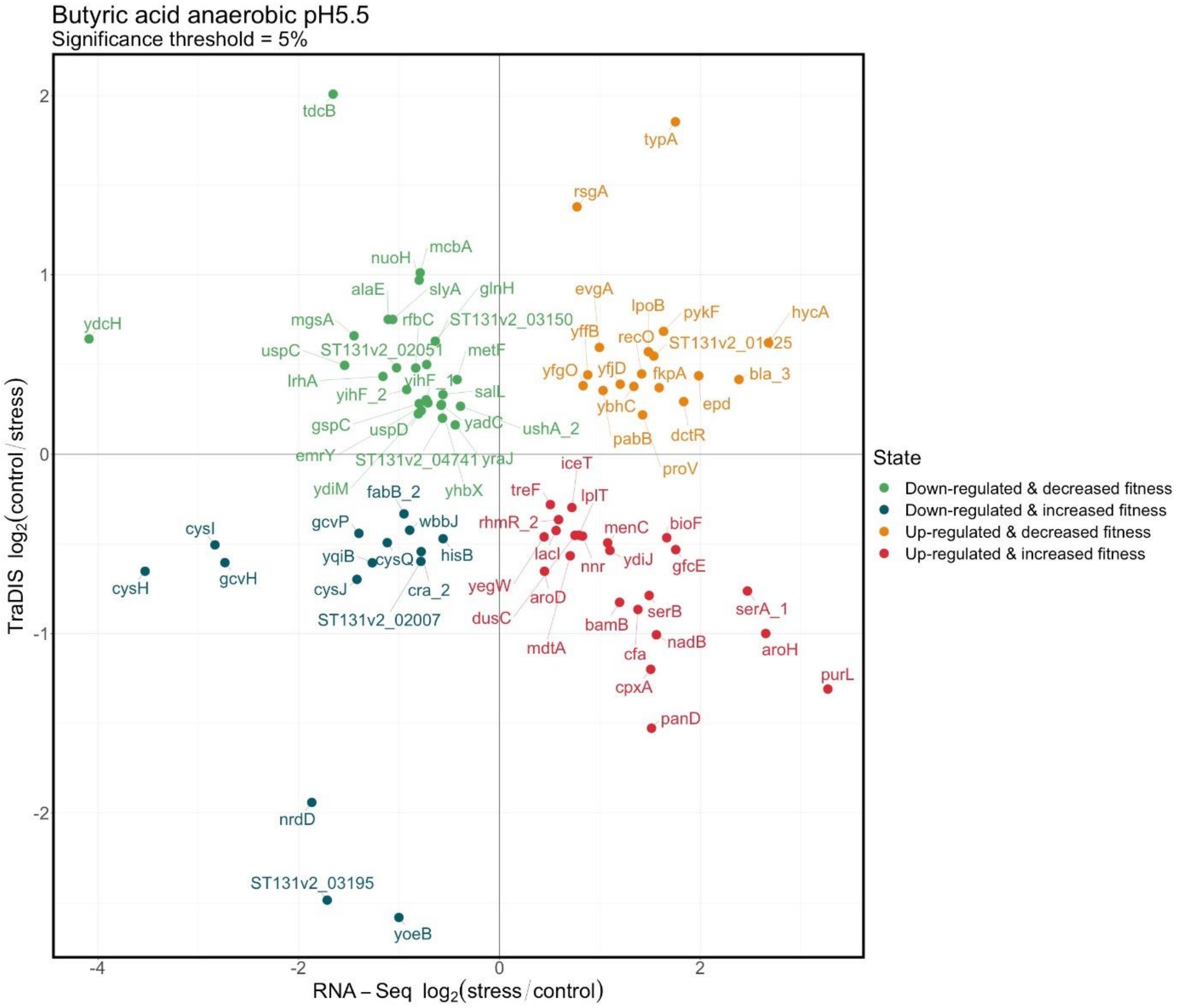
Butyric acid anaerobic pH5.5
Significance threshold = 5%
TraDIS log2(control/stress)
RNA – Seq log2(stress/control)
State
Down-regulated & decreased fitness
Down-regulated & increased fitness
Up-regulated & decreased fitness
Up-regulated & increased fitness
tdcB
ydcH
nuoH
mcbA
alaE
slyA
glnH
mgsA
rfbC
ST131v2_03150
uspC
ST131v2_02051
metF
lrhA
yihF_1
yihF_2
salL
gspC
uspD
yadC
ushA_2
emrY
ST131v2_04741
yraJ
ydiM
yhbX
typA
rsgA
evgA
lpoB
pykF
hycA
yffB
recO
ST131v2_01025
yfgO
yfjD
fkpA
bla_3
ybhC
epd
pabB
dctR
proV
cysI
cysH
gcvH
fabB_2
gcvP
wbbJ
yqiB
cysQ
hisB
cysJ
cra_2
ST131v2_02007
nrdD
ST131v2_03195
yoeB
iceT
treF
lplT
rhmR_2
menC
bioF
lacI
ydiJ
nnr
gfcE
yegW
aroD
dusC
mdtA
bamB
serB
serA_1
nadB
aroH
cfa
cpxA
purL
panD

Figure S7H

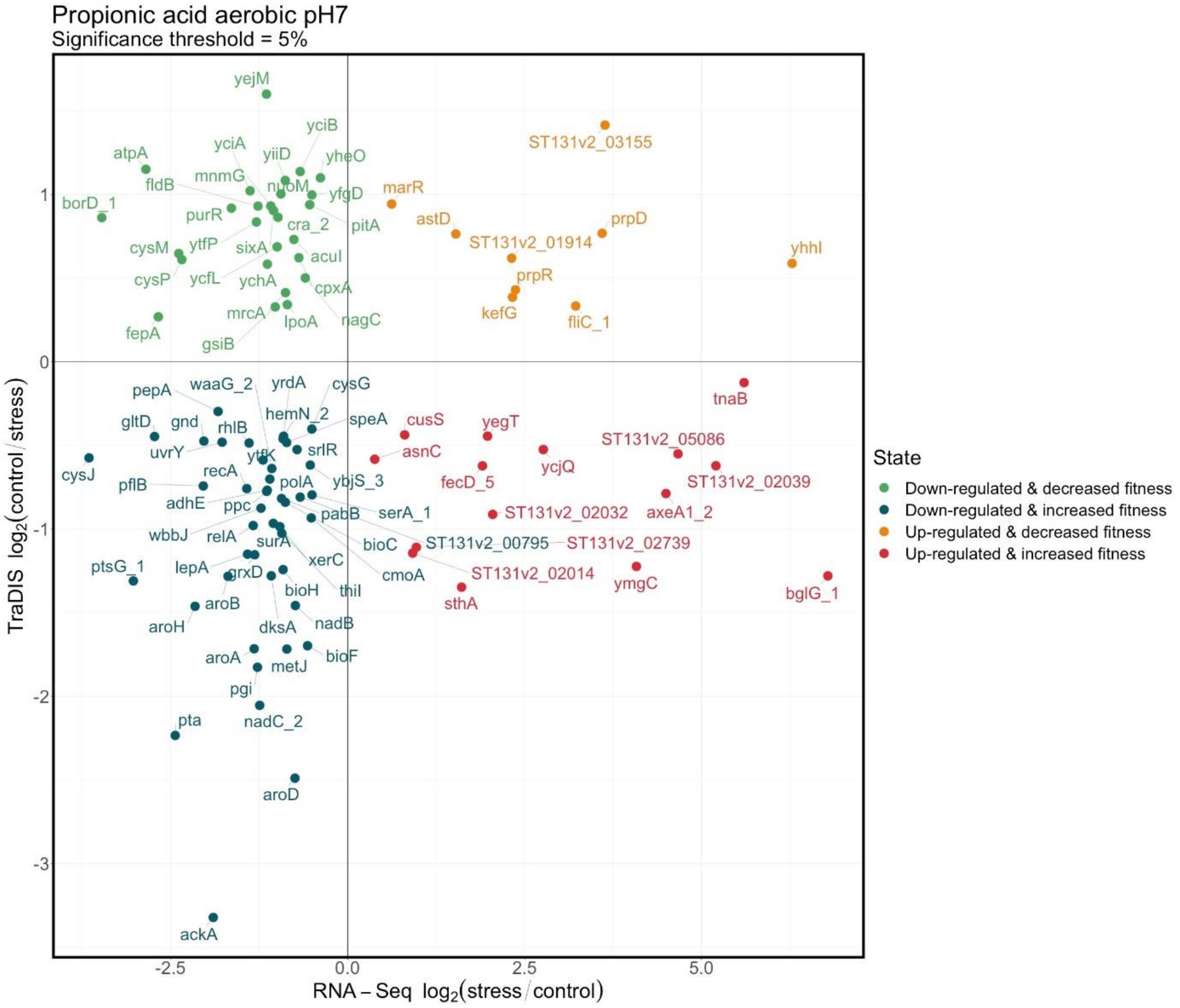
Propionic acid aerobic pH7
Significance threshold = 5%
TraDIS log2(control/stress)
RNA – Seq log2(stress/control)
State
Down-regulated & decreased fitness
Down-regulated & increased fitness
Up-regulated & decreased fitness
Up-regulated & increased fitness

Figure S7I

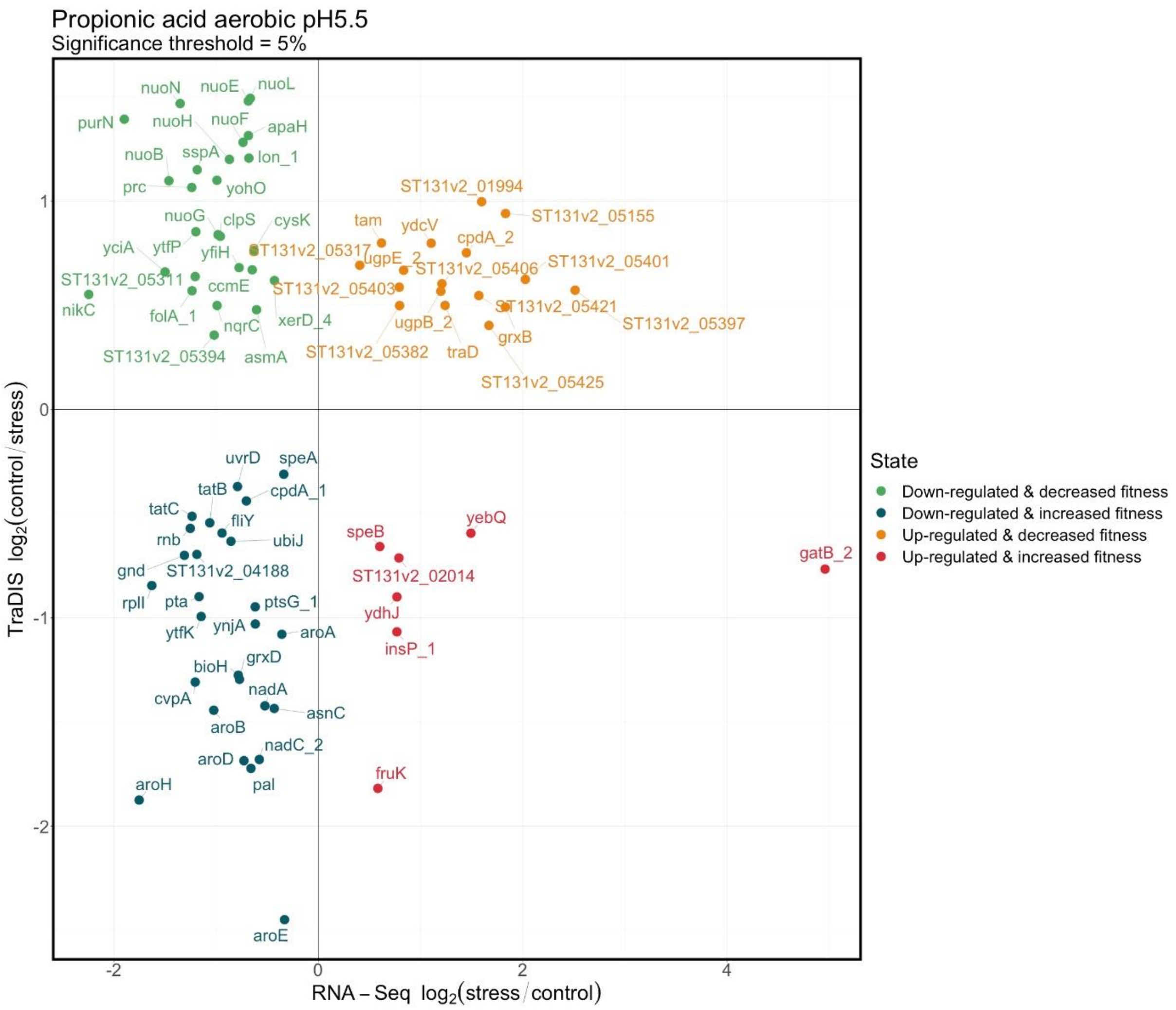

Propionic acid aerobic pH5.5
Significance threshold = 5%
TraDIS $\log_2(\text{control}/\text{stress})$
RNA – Seq $\log_2(\text{stress}/\text{control})$
State
Down-regulated & decreased fitness
Down-regulated & increased fitness
Up-regulated & decreased fitness
Up-regulated & increased fitness
nuoN
nuoE
nuoL
purN
nuoH
nuoF
apaH
nuoB
sspA
lon_1
prc
yohO
nuoG
clpS
cysK
yciA
ytfP
yfiH
ST131v2_05317
ST131v2_05311
ccmE
nikC
folA_1
nqrC
xerD_4
ST131v2_05394
asmA
ST131v2_01994
ST131v2_05155
tam
ydcV
cpdA_2
ugpE_2
ST131v2_05406
ST131v2_05401
ST131v2_05403
ST131v2_05421
ugpB_2
grxB
ST131v2_05397
ST131v2_05382
traD
ST131v2_05425
uvrD
speA
tatB
cpdA_1
tatC
fliY
rnb
ubiJ
gnd
ST131v2_04188
rplI
pta
ptsG_1
ytfK
ynjA
aroA
bioH
grxD
cvpA
nadA
asnC
aroB
nadC_2
aroD
pal
aroH
aroE
speB
yebQ
ST131v2_02014
ydhJ
insP_1
fruK
gatB_2

Figure S7J

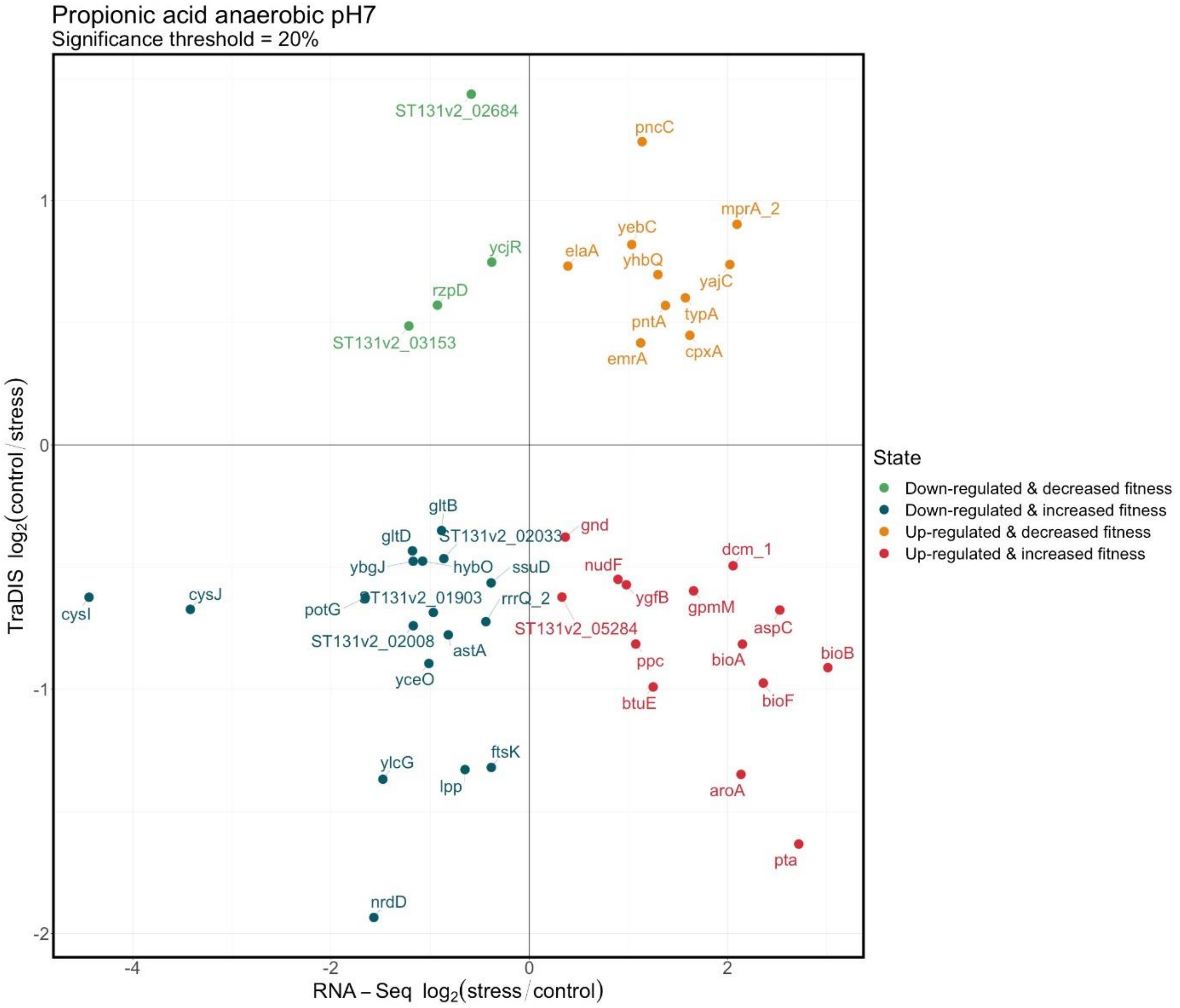

Propionic acid anaerobic pH7
Significance threshold = 20%
TraDIS log2(control/stress)
RNA – Seq log2(stress/control)
State
Down-regulated & decreased fitness
Down-regulated & increased fitness
Up-regulated & decreased fitness
Up-regulated & increased fitness
ST131v2_02684
pncC
mprA_2
yebC
elaA
yhbQ
ycjR
yajC
rzpD
typA
pntA
ST131v2_03153
cpxA
emrA
gltB
gnd
gltD
ST131v2_02033
dcm_1
nudF
ybgJ
hybO
ssuD
cysJ
ygfB
gpmM
cysI
potG
ST131v2_01903
rrrQ_2
aspC
ST131v2_05284
ST131v2_02008
astA
bioB
ppc
bioA
yceO
btuE
bioF
ftsK
ylcG
lpp
aroA
pta
nrdD

Figure S7K

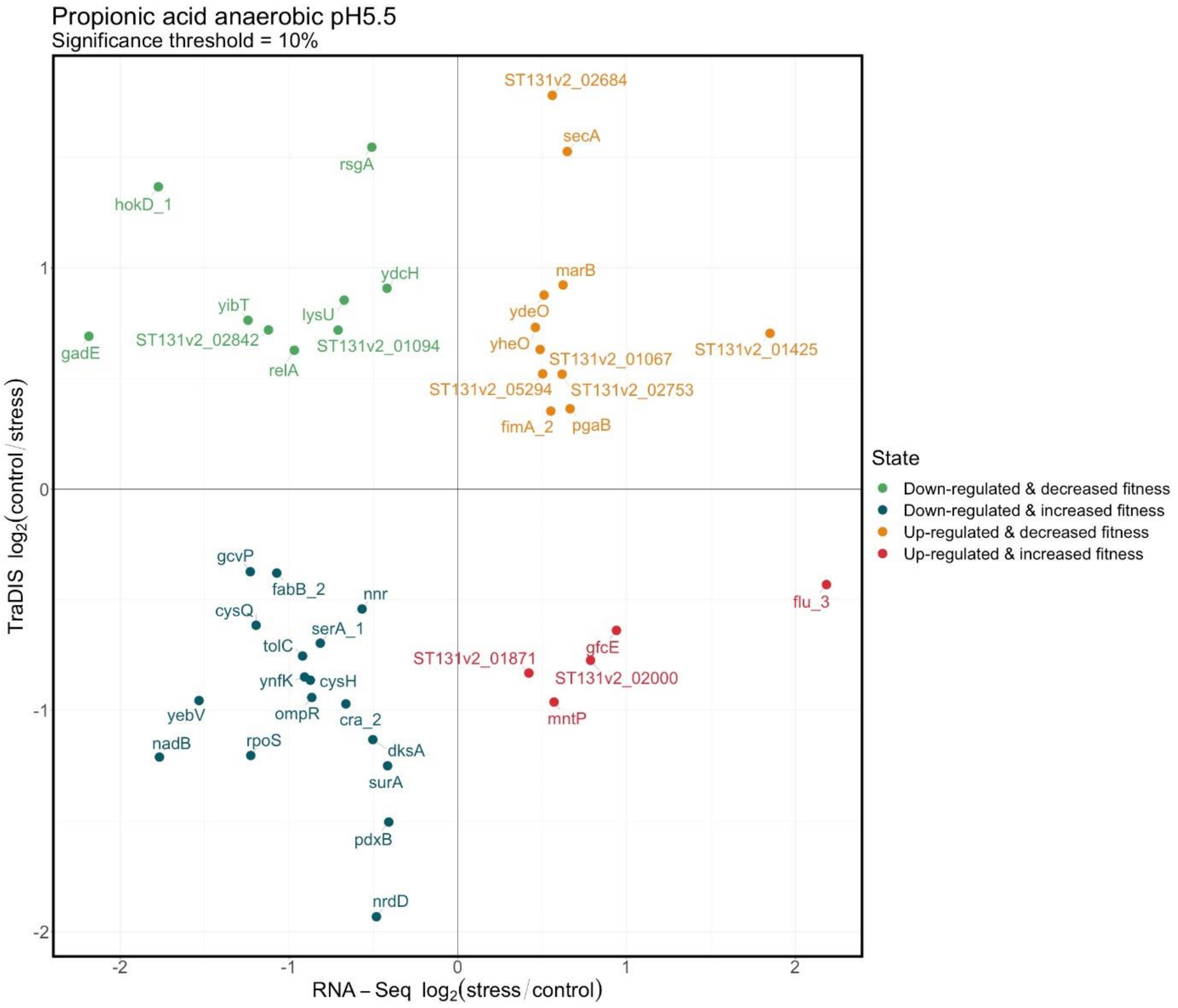

Propionic acid anaerobic pH5.5
Significance threshold = 10%
ST131v2_02684
secA
rsgA
hokD_1
ydcH
marB
yibT
lysU
ydeO
ST131v2_02842
ST131v2_01094
yheO
ST131v2_01425
gadE
relA
ST131v2_01067
ST131v2_05294
ST131v2_02753
fimA_2
pgaB
gcvP
fabB_2
nnr
cysQ
serA_1
tolC
ST131v2_01871
gfcE
ST131v2_02000
ynfK
cysH
ompR
yebV
cra_2
mntP
nadB
rpoS
dksA
surA
pdxB
nrdD
flu_3
TraDIS log2(control/stress)
RNA – Seq log2(stress/control)
State
Down-regulated & decreased fitness
Down-regulated & increased fitness
Up-regulated & decreased fitness
Up-regulated & increased fitness

Figure S7L

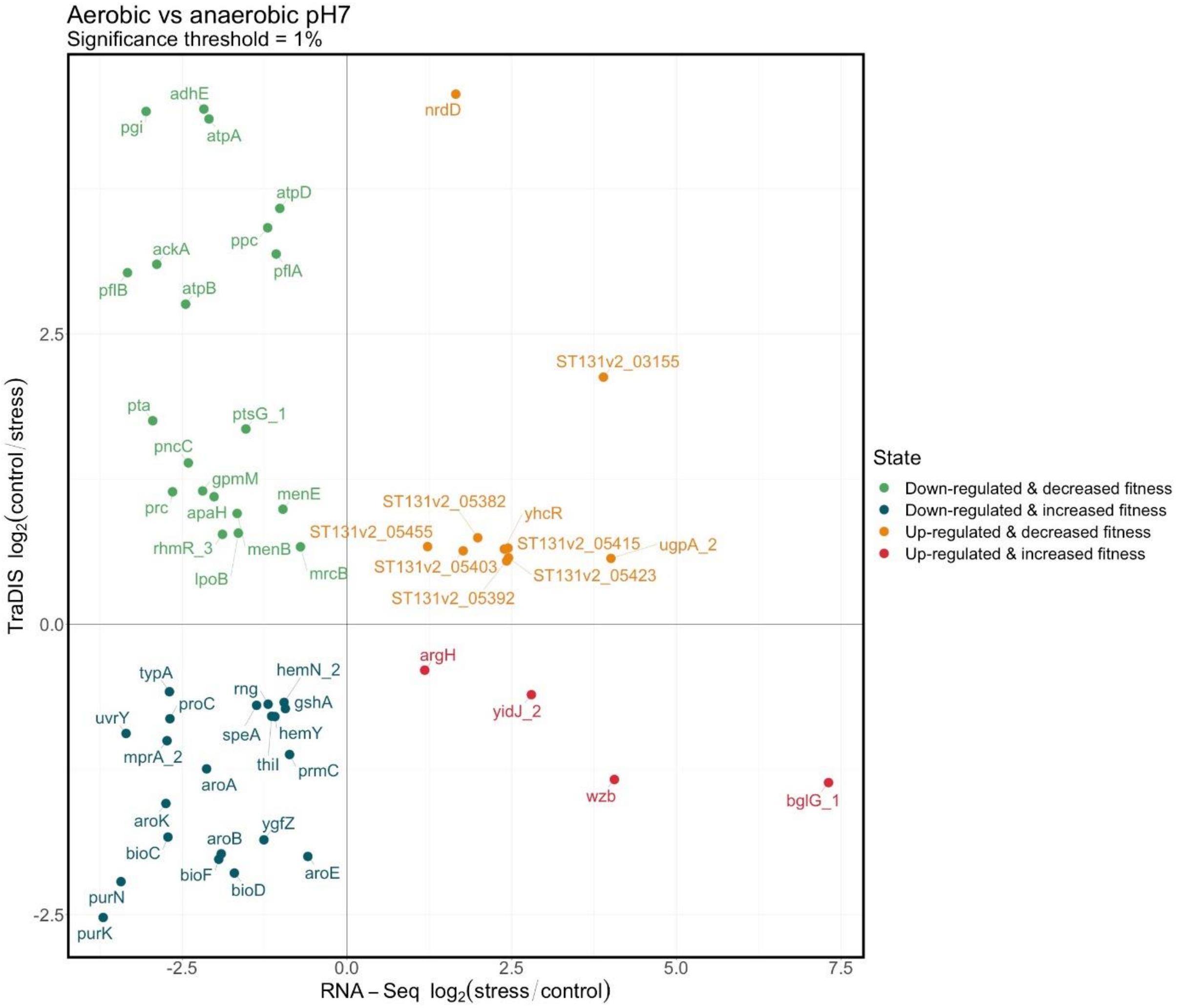

Aerobic vs anaerobic pH7
Significance threshold = 1%
TraDIS log2(control/stress)
RNA – Seq log2(stress/control)
2.5
0.0
-2.5
-2.5
0.0
2.5
5.0
7.5
adhE
pgi
atpA
atpD
ppc
ackA
pflA
pflB
atpB
pta
ptsG_1
pncC
gpmM
prc
menE
apaH
rhmR_3
menB
mrcB
lpoB
nrdD
ST131v2_03155
ST131v2_05382
yhcR
ST131v2_05455
ST131v2_05415
ugpA_2
ST131v2_05403
ST131v2_05423
ST131v2_05392
typA
hemN_2
rng
proC
gshA
uvrY
speA
hemY
mprA_2
thiI
prmC
aroA
aroK
ygfZ
aroB
bioC
bioF
aroE
bioD
purN
purK
argH
yidJ_2
wzb
bglG_1
State
Down-regulated & decreased fitness
Down-regulated & increased fitness
Up-regulated & decreased fitness
Up-regulated & increased fitness

Figure S7M

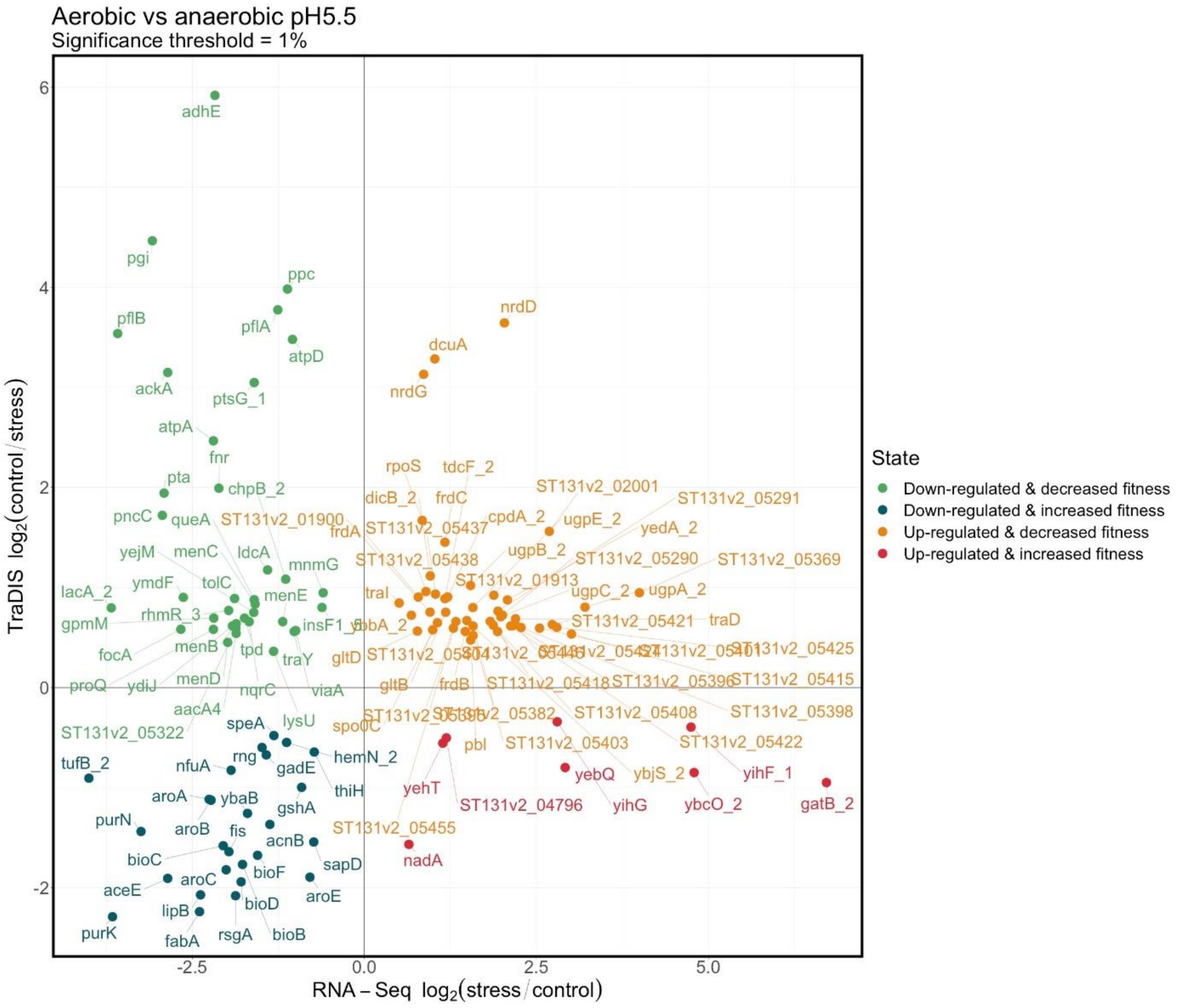

Aerobic vs anaerobic pH5.5
Significance threshold = 1%
TraDIS log2(control/stress)
RNA – Seq log2(stress/control)
6
4
2
0
-2
-2.5
0.0
2.5
5.0
State
Down-regulated & decreased fitness
Down-regulated & increased fitness
Up-regulated & decreased fitness
Up-regulated & increased fitness
adhE
pgi
ppc
pflB
pflA
atpD
ackA
ptsG_1
atpA
fnr
pta
chpB_2
pncC
queA
yejM
menC
ldcA
mnmG
ymdF
tolC
lacA_2
menE
gpmM
rhmR_3
insF1
focA
menB
tpd
traY
proQ
ydiJ
menD
nqrC
viaA
aacA4
ST131v2_05322
speA
lysU
tufB_2
nfuA
rng
gadE
hemN_2
thiH
aroA
ybaB
gshA
purN
aroB
fis
acnB
bioC
bioF
sapD
aceE
aroC
aroE
lipB
bioD
purK
fabA
rsgA
bioB
nrdD
dcuA
nrdG
rpoS
tdcF_2
ST131v2_02001
ST131v2_05291
dicB_2
frdC
ST131v2_01900
cpdA_2
ugpE_2
yedA_2
frdA
ST131v2_05437
ugpB_2
ST131v2_05290
ST131v2_05369
ST131v2_05438
ST131v2_01913
traI
ugpC_2
ugpA_2
ST131v2_05421
traD
gltD
ST131v2_05425
gltB
frdB
ST131v2_05418
ST131v2_05396
ST131v2_05415
ST131v2_05382
ST131v2_05408
ST131v2_05398
spo0C
pbl
ST131v2_05403
ST131v2_05422
yehT
yebQ
ybjS_2
yihF_1
ST131v2_04796
yihG
ybcO_2
gatB_2
ST131v2_05455
nadA

Figure S7N

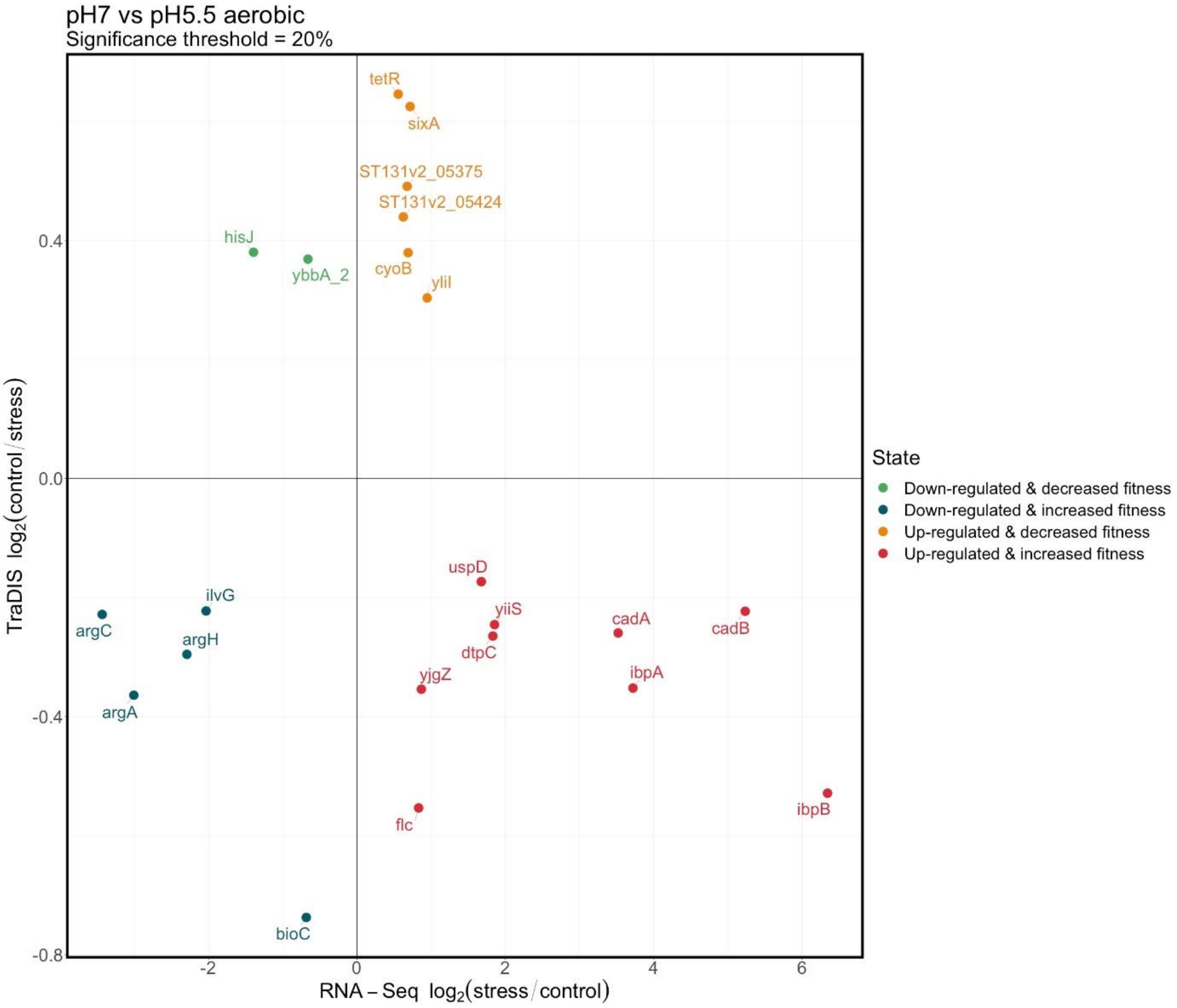

pH7 vs pH5.5 aerobic
Significance threshold = 20%
tetR
sixA
ST131v2_05375
ST131v2_05424
hisJ
ybbA_2
cyoB
yliI
uspD
ilvG
yiiS
argC
argH
dtpC
cadA
cadB
yjgZ
ibpA
argA
flc
ibpB
bioC
0.4
0.0
-0.4
-0.8
-2
0
2
4
6
TraDIS log2(control/stress)
RNA – Seq log2(stress/control)
State
Down-regulated & decreased fitness
Down-regulated & increased fitness
Up-regulated & decreased fitness
Up-regulated & increased fitness

Figure S7O

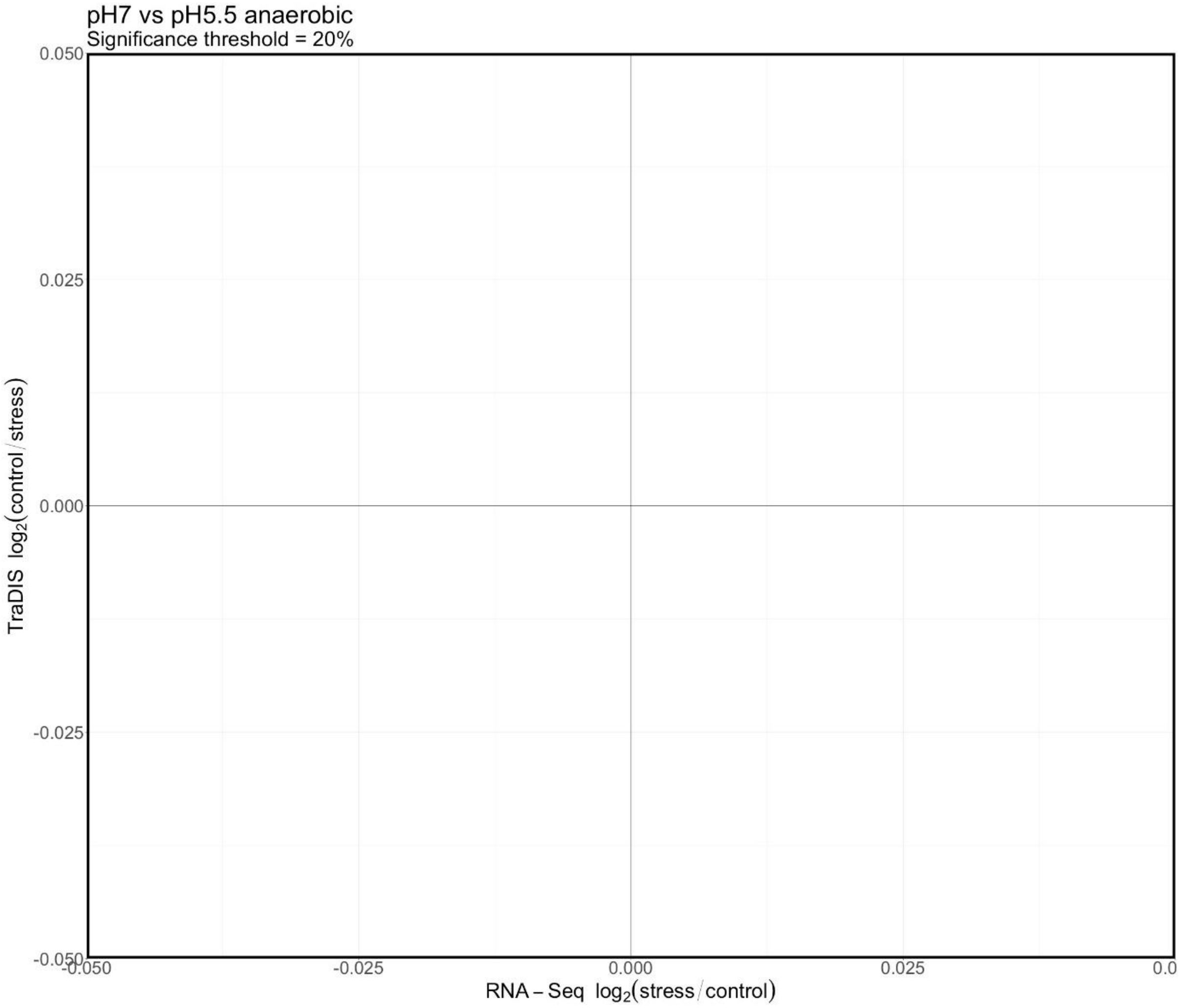

Figure S7P

Figure S7 Plots of RNAseq (stress/control, x-axis) vs traDIS (control/stress, y axis) for all conditions vs relevant controls, with colour coding to indicate whether gene expression is increased or decreased, and whether the mutants in the gene show overall higher or lower fitness. Only genes which are significant for both methods are shown. The specific condition and the significance threshold (false discovery rate) used for the plot is indicated on each graph. Note that the axes are not scaled the same in all figures. The final plot (Figure S7P) is blank because even at a FDR of 20%, no genes were significantly altered for both RNAseq and traDIS.

Figure S8A

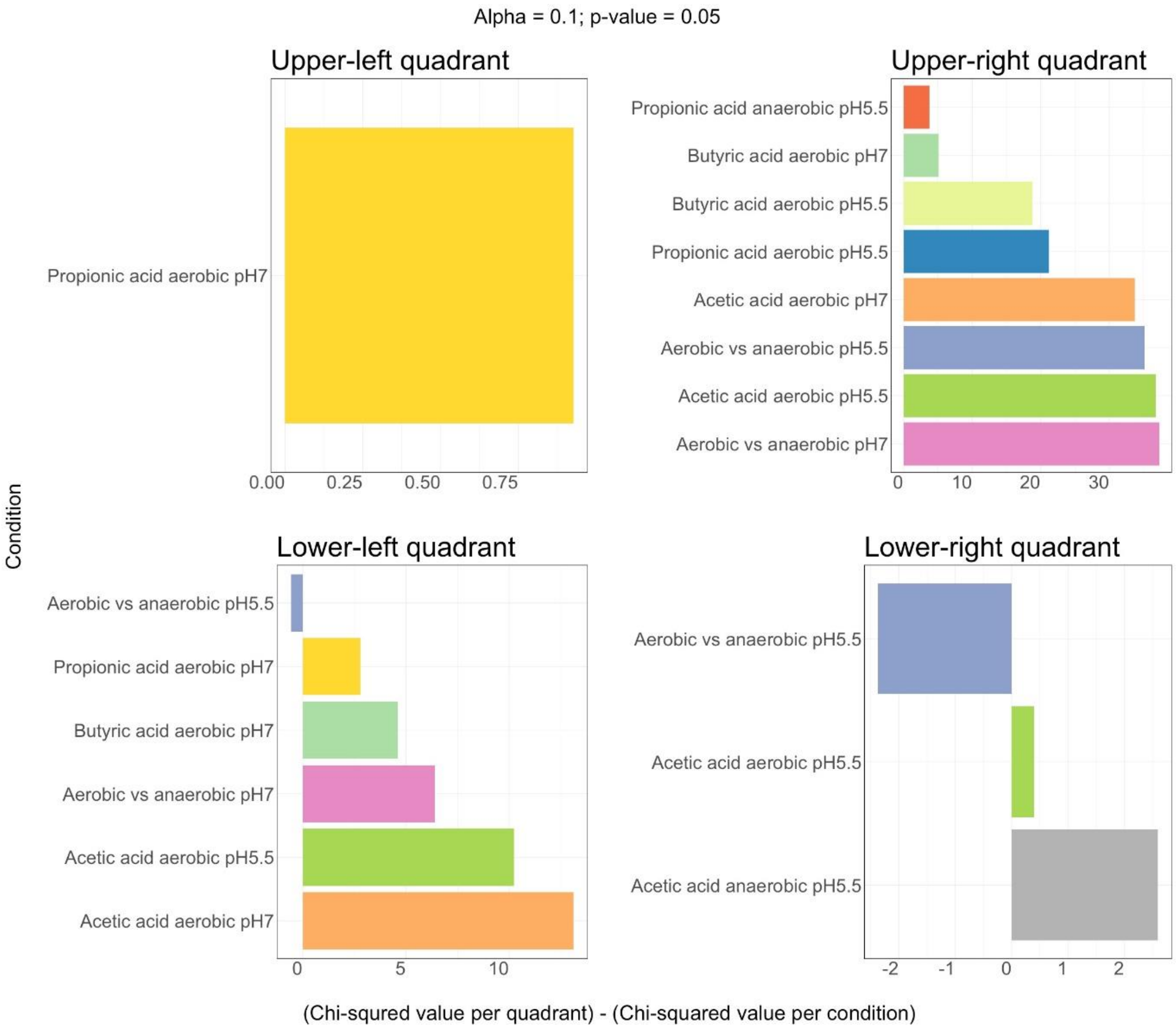
Alpha = 0.1; p-value = 0.05
Upper-left quadrant
Propionic acid aerobic pH7
0.00
0.25
0.50
0.75
Upper-right quadrant
Propionic acid anaerobic pH5.5
Butyric acid aerobic pH7
Butyric acid aerobic pH5.5
Propionic acid aerobic pH5.5
Acetic acid aerobic pH7
Aerobic vs anaerobic pH5.5
Acetic acid aerobic pH5.5
Aerobic vs anaerobic pH7
0
10
20
30
Condition
Lower-left quadrant
Aerobic vs anaerobic pH5.5
Propionic acid aerobic pH7
Butyric acid aerobic pH7
Aerobic vs anaerobic pH7
Acetic acid aerobic pH5.5
Acetic acid aerobic pH7
0
5
10
Lower-right quadrant
Aerobic vs anaerobic pH5.5
Acetic acid aerobic pH5.5
Acetic acid anaerobic pH5.5
-2
-1
0
1
2
(Chi-squred value per quadrant) - (Chi-squared value per condition)

Figure S8B

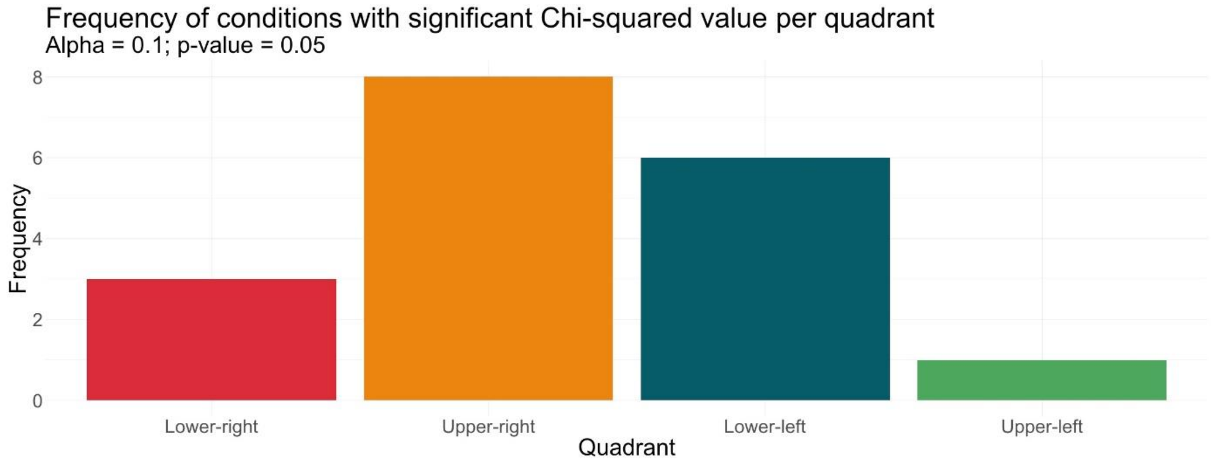

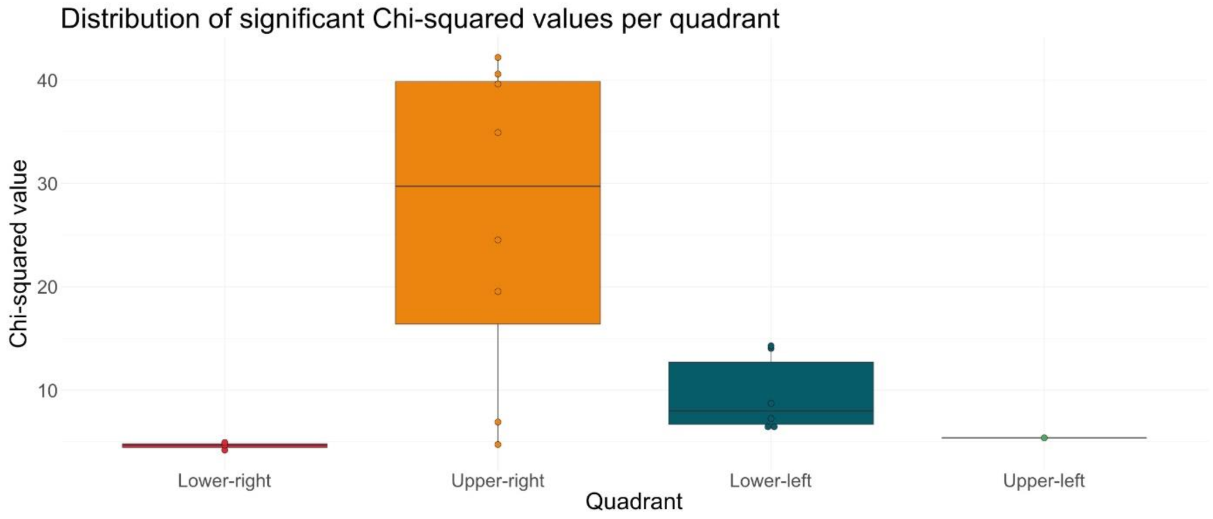

Figure S8: ST131-specific genes are enriched in the upper right and lower left quadrants of plots of RNAseq vs. traDIS data. Chi-squared values were calculated for all ST131-specific (i.e. non-*E. coli* core) genes for all conditions in all four quadrants; S7A shows conditions with a significant difference from the expected number of ST131 genes in the quadrant shown. The width of the bars has no significance in these diagrams. In all conditions except one (aerobic vs anaerobic cultures at pH 5.5) the ST131-specific genes were always seen at a greater frequency than expected by random chance. The total numbers of conditions with significant different levels of ST131-specific genes, and the individual and mean chi-squared values for all these conditions in each quadrant, are shown in Figure 7B. The straight lines in the centre of each box are the median, the upper and lower bounds (hinges) of the box are the 25th and 75th percentiles, and the dots represent the Chi-squared values for each condition.

Figure S9

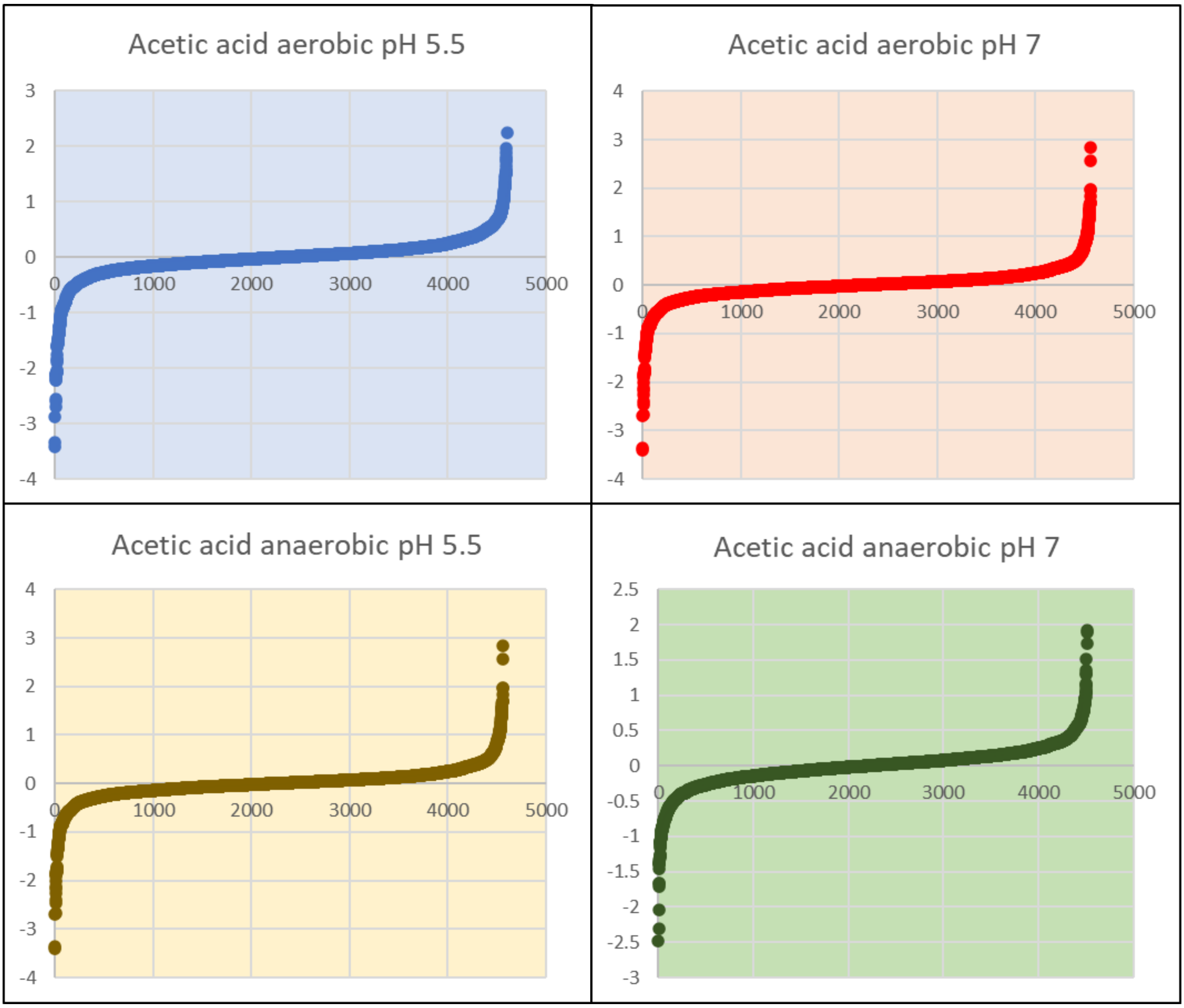

Figure S9: Gene fitness profiles for different conditions show the same profiles. For each of the four conditions shown, the log_2-fold change values for every gene were ranked and plotted in rank order, with the number in the order on the x-axis and the log_2-fold change values on the y axis. Note that the gene rank order is not the same in each condition.
