# Supplementary material for: Mapping the Transcriptional and Fitness Landscapes of a Pathogenic E. coli Strain: The Effects of Organic Acid Stress under Aerobic and Anaerobic Conditions
Source: Genes (Basel). 2020 Dec 31;12(1):53. doi: 10.3390/genes12010053 (PMC7824302; doi:10.3390/genes12010053)
Supplement: Supplementary file 1 [file genes-12-00053-s001.zip › genes-1011081 supplementary/Table_S2.docx]

Table S2: Genes which showed significant (adjusted P-value less than or equal to 0.05) alteration in pH-regulated expression under aerobic conditions I the absence of added organic acids.

| **Gene** | **logFC.x** | **Product** |
| --- | --- | --- |
| **up-regulated at pH 5.5. vs pH 7** | | |
| *ibpB* | 6.343744 | heat shock chaperone |
| *cadB* | 5.234526 | putative lysine/cadaverine transporter |
| *ibpA* | 3.721288 | heat shock chaperone |
| *cadA* | 3.5216 | lysine decarboxylase, acid-inducible |
| *cpxP* | 2.986065 | inhibitor of the cpx response; periplasmic adaptor protein |
| *lpxP* | 2.882668 | palmitoleoyl-acyl carrier protein (ACP)- dependent acyltransferase |
| *yeiH* | 2.787156 | UPF0324 family inner membrane protein |
| *macB_1* | 2.215671 | macrolide ABC transporter peremase/ATPase |
| *bssS* | 2.156666 | biofilm regulator |
| *csiD* | 2.064786 | carbon starvation protein |
| *clpB_2* | 1.909927 | protein disaggregation chaperone |
| *yeaY* | 1.854007 | Slp family lipoprotein, RpoE-regulated |
| **down-regulated at pH 5.5. vs pH 7** | | |
| *argD* | -3.67824 | bifunctional acetylornithine aminotransferase and succinyldiaminopimelate aminotransferase |
| *argC* | -3.43282 | N-acetyl-gamma-glutamylphosphate reductase, NAD(P)-binding |
| *artJ* | -3.29413 | arginine ABC transporter periplasmic binding protein |
| *argA* | -3.0066 | amino acid N-acetyltransferase and inactive acetylglutamate kinase |
| *argB* | -2.85731 | acetylglutamate kinase |
| *argH* | -2.29079 | argininosuccinate lyase |
| *ilvG* | -2.03282 | hypothetical protein |
